# Bacteriophage therapy as an alternative technique for treatment of multidrug-resistant bacteria causing diabetic foot infection

**DOI:** 10.1007/s10123-022-00293-2

**Published:** 2022-11-09

**Authors:** Amira Mohamed Ghanaim, Mohammed Abdulaziz Foaad, Eman Zakaria Gomaa, Khalid Abdelfatah El Dougdoug, Gamal Eldidamony Mohamed, Ahmed Hamed Arisha, Tarek Khamis

**Affiliations:** 1 Department of Biological and Geological Sciences, Faculty of Education, Ain Shams University, Cairo, Egypt; 2 Department of Microbiology, Faculty of Agriculture, Ain Shams University, Cairo, Egypt; 3 Department of Microbiology, Faculty of Science, Zagazig University, Zagazig, Egypt; 4 Department of Physiology, Faculty of Veterinary Medicine, Zagazig University, Zagazig, Egypt; 5 Department of Pharmacology, Faculty of Veterinary Medicine, Zagazig University, Zagazig, Egypt

**Keywords:** Diabetic foot ulcer, Multidrug-resistant bacteria, Bacteriophage therapy, Wound healing, Inflammatory, And anti-inflammatory markers

## Abstract

Diabetic foot ulcer (DFU) represented the most feared diabetic complication that caused the hospitalization of the diabetic patient. DFU was usually characterized with delayed healing as the diabetic neuropathy, angiopathy, and ulcer concomitant infections, among them, are multidrug-resistant (MDR) bacteria that emphasized the clinical importance for developing new therapeutic strategy with safe and effective alternatives for the antibiotics to overcome DFU-MDR bacterial infection. Bacteriophage therapy was considered a novel approach to eradicate the MDR, but its role in the polymicrobial infection of the DFU remains elusive. Thus, the current work was designed to investigate the effect of the topical application of the phage cocktail on the healing of the diabetic wound infected with clinical isolates of *Staphylococcus aureus*, *Pseudomonas aeruginosa*, *Klebsiella variicola*, *Escherichia coli*, and *Proteus mirabilis*. Bacterial isolation was performed from clinical hospitalized and non-hospitalized cases of DFU, identified morphologically, biochemically, molecularly via *16 s rRNA* sequencing, and typed for the antibiotic resistance pattern. Moreover, phages were isolated from the aforementioned clinical isolates and identified with electron microscope. Forty-five adult male Sprague–Dawley rats were assigned in 3 groups (15 rats each), namely, the diabetic infected wound group, diabetic infected wound ceftriaxone-treated group, and the diabetic infected wound phage cocktail-treated group. The results revealed that phage cocktail had a superior effect over the ceftriaxone in wound healing parameters (wound size, wound index, wound bacterial load, and mRNA expression); wound healing markers (*Cola1a*, *Fn1*, *MMP9*, *PCNA*, and *TGF-β*); inflammatory markers (*TNF-α*, *NF-κβ*, *IL-1β*, *IL-8*, and *MCP-1*); anti-inflammatory markers (*IL-10* and *IL-4*); and diabetic wound collagen deposition; and also the histomorphic picture of the diabetic infected wound. Based on the current findings, it could be speculated that phage therapy could be considered a novel antibiotic substitute in the DFU with MDR-polymicrobial infection therapeutic strategies.

**Supplementary Information:**

The online version contains supplementary material available at 10.1007/s10123-022-00293-2.

## Introduction

A diabetic foot is one of the most feared complications of diabetes that causes hospitalization among diabetic patients. It is characterized by several pathological complications such as neuropathy, peripheral vascular disease, foot ulceration, and infection with or without osteomyelitis, which increase the risk of gangrene development that eventually leads to limb amputation (Pondei et al. 2013; Pallavali et al. 2017). According to the Center for Disease Control and Prevention (CDC), Egypt is among the 10 top countries with the highest prevalence of diabetes, with the 9th rank worldwide, where there are 7.5 million diabetic patients, 15% of whom have developed a diabetic foot ulcer (DFU) (CDC 2013).

DFU is frequently associated with multidrug-resistant bacteria infections caused by either Gram-positive bacteria like *Staphylococcus aureus*, *Staphylococcus epidermidis*, *Enterococcus* spp., and *Streptococcus* spp. or Gram-negative ones such as *Pseudomonas* spp*.*, *Escherichia coli*, *Enterobacter* spp., *Acinetobacter baumannii*, *Citrobacter* spp., *Bacteroides* spp*.*, *Peptostreptococcus* spp., *Fusobacterium* spp*.*, and *Clostridium* spp. (approximately 40–80% of the DFU patients) (Sanchez et al. 2013; Noor et al. 2015; Mahgoub et al. 2015; Castillo et al. 2019).

The emergence of multidrug-resistant (MDR) microorganisms, which cause antibiotic regimen therapy for DFU to fail, has highlighted the clinical importance of developing valid alternative therapeutic strategies (Maciejewska et al. 2018; Cha et al. 2018). Phage therapy was a promising strategy for DFU because it could reduce or eliminate DFU-MDR bacterial infections while providing several advantages over conventional antimicrobial drugs, including specificity, stability in harsh environmental conditions, and a lack of the cytotoxicity against keratinocytes and fibroblasts that is experienced with antimicrobial molecules since the phage do not infect eukaryotic cells. Furthermore, it replicated at the site of the infection, providing a dynamic therapeutic strategy (Butler et al. 2017; Roach and Debarbieux 2017).

Although PT has become a viable preserving technology for agricultural and food applications, its application for DFU-MDR polymicrobial infection remains elusive (Bolocan et al. 2016; El-Telbany et al. 2021). Therefore, the current study was designed to determine the bacterial profile of infected diabetic foot ulcers and the antibiotic resistance pattern of the bacterial isolates and to investigate the phage therapeutic potency against an excisional diabetic wound model infected with polymicrobial clinical isolates in type 1 diabetic rats.

## Material and methods

### Collection of specimens and bacterial isolation

A total of 85 selected DFI specimens were obtained from hospitalized DFU patients that received the antimicrobial therapeutic strategy with one or more antibiotics: vancomycin, clindamycin, imipenem, ciprofloxacin, ceftriaxone, cefotax, amoxicillin-clavulanate, levofloxacin, cephalexin, meropenem, cefepime, and ampicillin-sulbactam, in the Al Demerdash Hospital, Nasr City Health Insurance hospital, Ramses Health Insurance hospital, and diabetic foot clinics from December 2017 to May 2018, under aseptic conditions with written informed consent, then delivered to the microbiology lab within 1 h, cultured on blood and MacConkey agar (oxoid) using the plate streaking technique, and incubated at 35–37 °C for 24–48 h (Akhi et al. 2013). The isolated bacterial colonies were chosen and picked up according to culture characteristics (color, form, elevation, opacity, texture, and margin), then purified by successive subculturing on the same media as appropriate and stored at 4 °C till used.

### The antibiotic resistance pattern for the clinical isolates

The antibiotic susceptibility pattern of the clinical isolates was performed according to the Kirby-Bauer disc diffusion method (Bauer et al. 1966). In this assay, all clinical isolates were tested for 25 antibiotic discs (Oxoid, UK) of different groups. In brief, Muller-Hinton plates were inoculated with 0.1 ml of each bacterial culture adjusted to OD_600_ = 0.6 (10^8^ CFU/ml). Then, the antibiotic discs were aseptically placed on the surface and incubated at 35 °C for 24 h. The diameter of the inhibition zone was measured in millimeters (mm) and interpreted as sensitive (S), intermediate (I), or resistant (R) according to interpretative criteria for antimicrobial susceptible testing, and a multiple resistant index was calculated according to Krumperman (1983). Multidrug-resistant clinical isolates with high MRI were selected for identification using Vitek2 in the Animal Health Research Institute, Giza, and confirmed genetically using 16S rRNA gene analysis (Funke et al. 1998).

### Identification of MDR clinical isolates using 16S rRNA gene

After DNA extraction, PCR amplification was performed to confirm the identity of the selected MDR isolates using universal 1492r primer (5′-TACCTTGTTACGACTT) (forward primer: 5′-AGAGTTTGATCCTGGCTCAG-3′ and reverse primer: 5′-GGTTACCTTGTTACGACTT-3′). The PCR product was run and visualized by loading 10 μl of the PCR product per lane on a 1.8% agarose gel (Gomhuria Company, Egypt) that was stained with ethidium bromide against a 100-bp DNA ladder as a marker, using 1X TAE as a running buffer, and photographed by a gel documentation system to identify its size. PCR products were purified according to the manufacture procedure of Gene JET PCR Purification Kit (Thermo Scientific, Cat. No. K0701) and sequenced using the standard Sanger method on an ABI 3730XL DNA Sequencer at Macrogen sequencing services (Macrogen, Seoul, South Korea, for forward and reverse sequencing). The sequence was submitted to the Gene Bank at http://blast.ncbi.nlm.neh.gov using the Basic Local Alignment Search Tool Program and a cluster analysis and phylogenetic tree were constructed. The Gene Bank nucleotide sequence accession numbers for partial sequences of the 16S rRNA gene were generated in this study (Altschul et al. 1990).

### Bacteriophage isolation and enrichment

Three sewage samples were collected from sewage water and tested for the incidence of phages. Isolation and enrichment of the desired phages were performed according to Adams (1959). After the centrifugation and purification of the collected samples, they were transferred to a conical flask containing nutrient broth and inoculated with the tested bacteria: *Staphylococcus aureus*, *Pseudomonas aeruginosa*, *Klebsiella variicola*, *Escherichia coli*, and *Proteus mirabilis* (OD_600_ = 0.4–0.6). Flasks were incubated at 37 °C for 24 h with shaking at 100 rpm and then centrifuged at 6000 × g for 10 min. The supernatant of each sample was filtered through a 0.45-μm pore size cellulose acetate syringe filter to remove any bacterial cells that may be present and stored at 4 °C. The obtained crude lysate of the phages was assayed qualitatively by spot test and quantitatively by plaque assay and expressed as plaque-forming units (PFU/ml) according to Adams (1959).

### Transmission electron microscopy

The morphological properties of isolated phages were determined using a transmission electron microscope (TEM) as described by Bradley (1967). One drop of each purified phage with high titers was placed on a 400-mesh carbon-coated cupper grid for 10 s and examined using a Hitachi H600A electron microscope at 80 kV in the Electron Microscopy Unit at the Faculty of Agriculture, Mansoura University, Egypt.

### Thermal and pH stability for the isolated phages

Thermal inactivation point of phages in vitro was carried out by exposure of phages to different degrees of temperature: 40, 50, 60, 70, 80, and 90 °C for 10 min using a water bath and then immediately cooled under tap water. The treated phage was diluted and assayed by the plaque assay according to Philipson et al. (1960). In addition, the ability of the phages to survive at different pH levels was evaluated by exposing the phage suspension to different pH values from 2 to 12 using 0.1 M HCl/NaOH over 1 h at 37 °C. The stability of survival was checked qualitatively and quantitatively by using the plaque assay (Dhar et al.^1978^).

### Phage therapeutic studies

The therapeutic potential of bacteriophages specific for MDR *S. aureus*, *P. aeruginosa*, *K*. *variicola*, *P*. *mirabilis*, and *E*. *coli* was evaluated for their abilities to treat experimental foot infection in diabetic rats.

### Preparation of phage cocktail

The S2, Ps1, K4, C3, and Pr2 phage cocktails were prepared by adding equal volumes of each individual purified phage solution at 10^9^ PFU/ml and stored at 4 °C until needed.

### Lab animal

Forty-five male adult mature Sprague–Dawley rats 8–10 weeks old and weighing 250–300 g were purchased from Lab. Animal House, Faculty of Veterinary Medicine, Zagazig University. Rats were housed separately in polypropylene rat cages under standard conditions, including 45:50 relative humidity, 12-h light/dark cycle, and a temperature of 22 ± 2 °C. Rats were fed on a standard pelleted diet ad libitum with free access to water throughout the experimental period. For 1 week before any experimental procedures, lab animals were left for acclimatization. All experimental procedures were done in accordance with the Institutional Animal Care and Use Committee (IACUC), ARRIVE guidelines, and the National Institutes of Health guide for the care and use of laboratory animals.

### Toxicity testing of phage cocktail in rats

The toxicity of phage cocktail suspension was investigated in rats according to the method of Soothill (1992). Six adult male rats were injected intraperitoneally with 0.25 ml of phage cocktail suspension (10^12^ PFU/ml). Three uninjected rats were retained as controls. The rats were observed for signs of illness, and their rectal temperatures were taken hourly during the first 5 h after injection and then daily for 4 days.

### Induction of type 1 diabetes

Type 1 diabetes was induced in the rats using streptozotocin (STZ) at a dose of 65 mg/kg body weight according to the method described by King (2012).

### Creation of diabetic wound and experimental design

A diabetic wound was created according to the method described by Muhammad et al. (2016). After 1 week post validation of type 1 diabetes, diabetic wounded rats were divided into three groups each of 15 rats: group (1) diabetic wound infected with bacteria (2 × 10^8^ CFU/ml); group (2) diabetic wound infected and topically treated with 0.5 ml of 2% ceftriaxone (Zakaria et al. 2016) 2 days post diabetic wound induction for 5 consecutive days; and group (3) diabetic wound infected and treated topically with a 5-phage cocktail at a dose of 10^9^ PFU/ml (MOI-10) 2 days post diabetic wound induction for 5 consecutive days (Chhibber et al. 2018). Rats were kept separately in polypropylene cages to avoid fighting and wound biting. Then the wound diameter and wound healing index were evaluated at 3-day intervals till 21 days. At the end of 21 days, blood was collected from the retro-orbital eye saphenous. Then, rats were sacrificed and the skin of the wound was excised and divided into four parts: the first part was collected on 10% formalin neutral buffer for histopathological examination; the second part was collected on phosphate-buffered saline (PBS) for determining wound microbial load; the third part was stored at – 20 °C for oxidant/antioxidant activity; and the fourth part, 50 mg of diabetic skin wound was collected on 1 ml of quiazol (Quiagen, Germany) for total RNA extraction for qRT-PCR to quantify relative diabetic wound gene expression.

### Measuring of glycemic parameters

Blood glucose was measured 7 days post STZ injection and 21 days post diabetic wound induction with a digital glucometer (U-Right, Korea). Insulin was also measured in serum with the ELISA Kit (SunRedBio, China) according to the method previously described by Solarek et al. (2019). The lipid peroxidation marker malondialdehyde (MDA) and antioxidant enzymes glutathione peroxidase (GPx), catalase (CAT), and superoxide dismutase (SOD) were locally measured with a sandwich ELISA kit (SunRedBio, China) according to the method developed by Zhang et al. (2019). Bacterial load was assessed according to the method of Park et al. (2009).

### Quantitative reverse transcription PCR (RT-qPCR) for wound gene expression

Total RNA was extracted from 50 mg of skin tissue with 1 ml of quiazol (Quiagen, Germany) according to the manufacturer’s instructions. The extracted total RNA was measured with an anoDrop® ND-1000 UV–Vis Spectrophotometer (Thermo Scientific, Waltham, MA, USA) at wavelengths of 260 and 280. Also, the ratio of OD 260/280 was calculated to determine the quality of the extracted RNA once that accepted for gene expression is located in the value between 1.8:2. Complementary DNA (cDNA) was synthesized from 1 μg with a high-capacity reverse transcriptase kit (Applied Biosystem, Foster City, CA, USA) in a final reaction volume of 20 μl (10 μl master mix and 10 μl RNA sample containing 1 μg RNA). For the qPCR reaction, cDNA was diluted to 1:10 and stored in aliquots at − 20 °C. The qPCR was done by a real-time thermal cycler, Rotor-Gene Q2 plex (Quiagen, Germany), according to the method described by Khamis et al. (2020). In brief, with a final volume reaction of 20 μl using TOPreal syberGreen (Enzynomics, Korea), 10 μl and 1 μl of each forward and reverse primer synthesized by Sangon Biotech (Beijing, China) (Sup. 1), 1 μl of 1:10 diluted cDNA, and up to 20 μl of nuclease free water with cycling conditions of initial denaturation at 95 °C for 10 min, 40 cycles of denaturation at 95 °C for 10 s, annealing at 60 °C for 15 s, and extension at 72 °C for 15 s, and melt curve analysis. The gene expression was measured as a relative fold change to the internal control reference gene (*Gapdh*) according to the method previously developed by Schmittgen and Livak (2008). In brief, Δct was calculated as the ct difference between the target gene and reference gene; then, ΔΔct was calculated as the difference between Δct of the sample and the average Δct of the control; finally, fold gene of the gene expression was calculated as 2^−(ΔΔct)^.

### Histopathological examination

The histopathological examination was done in accordance with Bancroft and Layton (2019). In brief, at the end of the experiment, diabetic rats were scarified and the skin of the wound area was rapidly removed and collected in 10% formalin neutral buffer, then embedded in a paraffin wax block and cut into 4-μm slices mounted on a microscope slide that was used for histological stains. Before any histopathological procedures, the slides were incubated in xylene and passaged in ethanol with different concentrations for rehydration and paraffin wax removal. Then, the slides were stained with hemotoxylin (H) and eosin (E) for assessment of wound healing and Masson’s trichome for collagen deposition (% of collagen deposition to control group was analyzed with Fiji software (http://fiji.sc)).

### Statistical analysis

Statistical analysis was performed by GraphPad Prism 8 software (GraphPad Software Inc., San Diego, CA, USA). The data is expressed as mean_standard error mean (SEM). Statistical comparisons were performed using a one-way analysis of variance (ANOVA) test followed by a post hoc Tukey test. The results indicated statistical significance when *P* < 0.05.

## Results

In the present study, 85 samples were obtained from diabetic foot patients. The majority of the specimens (88.2%) were obtained from hospitalized diabetic patients, but only 11.8% were obtained from clinics. Sixty-eight male (76.4%) and nineteen female (22.35%) diabetic cases with an age range of 40 to 80 years were included. The maximum number of patients (57.64%) was in the age group of 60 to 69 years, followed by the age group between 50 and 59 years (34.1%), and then the group between 70 and 80 (4.7%). On the other hand, the minimum number of patients was recorded in the age group of 40 to 49 years (2.3%). The percentage of amputation in patients was recorded and represented 42.4%. From 85 collected wound swab specimens, only 78 (91.8%) were positive for bacterial isolation, with an average of 2.6 organisms per ulcer. From 78 positive culture cases, 10 patients (12.8%) had monomicrobial infections and 68 patients (87.2%) had polymicrobial infections. In this study, a total of 208 bacterial isolates were obtained, and the proportion of Gram-negative bacilli was higher than Gram-positive cocci. The commonest isolate was *Staphylococcus* spp. (24%), followed by *Pseudomonas aeruginosa* (18.2%), *Klebsiella* sp*.* (17.3), *Proteus* sp*.* (15.6%), and *Escherichia coli* (12.9%), whereas the lowest prevalence was recorded for *Acinetobacter* sp*.* (4.8%), *Enterococcus* sp. (4.8%), and *Enterobacter* sp*.* (2.4%).

### Antibiotic resistance pattern of the isolates

Results of Table 1 showed that the antimicrobial susceptibility pattern showed that all *S. aureus* isolates were susceptible to vancomycin and linezolid but resistant to cloxacillin, ampicillin, penicillin, and cephradine with percentages of 92, 90, 84, and 84%, respectively. The antimicrobial resistance pattern of *Enterococcus* sp. was as follows: cephradine (100%), cloxacillin, ampicillin, and penicillin (80%), vancomycin (70%), and linezolid (0%). On the other hand, *P*. *aeruginosa*, *Klebsiella* sp., *Proteus* sp., *E*. *coli*, *Acinetobacter* sp., and *Enterobacter* sp. showed more than 70% resistance to ampicillin, more than 60% to carbencillin, more than 50% to norfloxacin, and the 1st, 2nd, and 3rd generation of cephalosporins except ceftriaxone. In addition, these isolates showed 40 to 63% sensitive to amikacin, except *Acinetobacter* sp. and *Proteus* sp. showed 81.5% to polymyxin B and colistin.

**Table 1:** The antibiotic resistance patterns of the Gram-negative and Gram-positive bacteria (% of resistance)

Antibiotic classes	Antibiotics	*S. aureus*	*Enterobacter*	*Pseudomonas*	*Klebsiella*	*Proteus*	*E. coli*	*Acinetobacter*	*Enterobacter*
B-lactams	Ampicillin	90	80	92.1	72.2	87.5	85.2	90	100
	Carbenicillin	NT	NT	68.4	80.5	75	63	90	80
	Cefoperazone Sulbactam	NT	NT	63.1	58.3	68.7	66.6	70	60
	Penicillin	84	80	NT	NT	NT	NT	NT	NT
	Cloxacillin	92	80	NT	NT	NT	NT	NT	NT
Monobactams	Aztreonam	NT	NT	55.3	66.6	78.1	63	90	60
Cephalosporins I	Cefazolin	NT	NT	68.4	75	68.7	85.2	90	80
	Cephradine	84	100	NT	NT	NT	NT	NT	NT
Cephalosporins II	Cefuroxime	NT	NT	68.4	72.2	78.1	81.5	90	80
	Cefoxitin	8	100	NT	NT	NT	NT	NT	NT
Cephalosporins III	Cefotaxime	68	30	55.3	63.8	81.2	70	70	100
	Ceftriaxone	20	80	26.3	25	28	29.6	80	60
	Cefepime	NT	NT	57.9	63.8	62.5	48.1	70	80
	Ceftazidime	NT	NT	65.7	69.4	81.2	85.1	90	80
Aminoglycosides	Tobramycin	NT	NT	42.1	63.8	59.4	44.4	60	60
	Amikacin	NT	NT	36.8	41.6	37.5	55.5	90	60
	Gentamicin	58	30	63.1	41.6	40.6	29.6	70	40
	Netilmicin	42	30	NT	NT	NT	NT	NT	NT
Quinolones	Ciprofloxacin	46	70	22.2	47.2	68.7	63	50	100
	Ofloxacin	NT	NT	47.4	61.1	59.4	74	50	80
	Norfloxacin	54	60	34.2	52.1	62.5	85.2	70	100
Carbapenems	Meropenem	NT	NT	44.7	58.3	43.7	25.9	70	60
Chloramphenicol	Chloramphenicol	56	80	68.4	47.2	53.1	51.8	50	100
Polymyxins	Polymyxin B	NT	NT	15.7	30.5	87.5	18.5	0	0
	Colistin	NT	NT	10.5	36.1	87.5	18.5	0	0
Sulfonamide	Trimetho-sulfamethazole1	54	80	47.4	72.2	68.7	81.5	70	80
Fluoroquinolones	Nalidix	66	80	55.3	75	68.7	63	50	60
Tetracyclines	Doxycycline	34	30	42.1	50	59.4	48.1	40	40
	Tetracycline	44	60	63.1	47.2	68.7	63	40	60
Nitrofurans	Nitrofuration	28	30	52.6	50	50	51.8	40	80
Macrolides	Erythromycin	58	100	NT	NT	NT	NT	NT	NT
	Azithromycin	32	20	NT	NT	NT	NT	NT	NT
Lincosamides	Clindamycin	56	40	NT	NT	NT	NT	NT	NT
Ansamycin	Rifampicin	74	50	NT	NT	NT	NT	NT	NT
Glycopeptides	Vancomycin	0	70	NT	NT	NT	NT	NT	NT
	Teicoplanin	66	70	NT	NT	NT	NT	NT	NT
Oxazolidinones	Linezolid	0	0	NT	NT	NT	NT	NT	NT
No. of isolates		50	10	38	36	32	27	10	5

*NT* not determined

The MDR isolates SP7a, SP15a, SP15c, SP20a, DP36c, DP38a, DP42a, and NCP60c were identified by a Viteck-2 system and were shown to be strains belonging to *Acinetobacter baumannii*, *Staphylococcus aureus*, *Escherichia coli*, *Enterococcus faecalis*, *Enterobacter cloacae*, *Klebsiella pneumonia* ssp. *pneumonia*, *Pseudomonas aeruginosa*, and *Proteus mirabilis*, respectively (Sup. 2). Five more prevalent MDR bacterial isolates (SP15a, DP42a, DP38a, SP15c, and NCP60c) with the highest index identified by Viteck-2 were selected for molecular confirmation using 16S rRNA analysis. The Gene Bank nucleotide sequence accession numbers for partial sequences of 16S rRNA gene were generated in this study using NCBI and recorded as *Staphylococcus aureus* SaEg01 LC596095.1, *Pseudomonas aeruginosa* AG01 LC586427, *Klebsiella variicola* KvEG01 LC589614, *Proteus mirabilis* PmEG01 LC589616, and *Escherichia coli* EcEG01 LC589615 (Sup. 3).

### Bacteriophage features

**Fig. 1:**
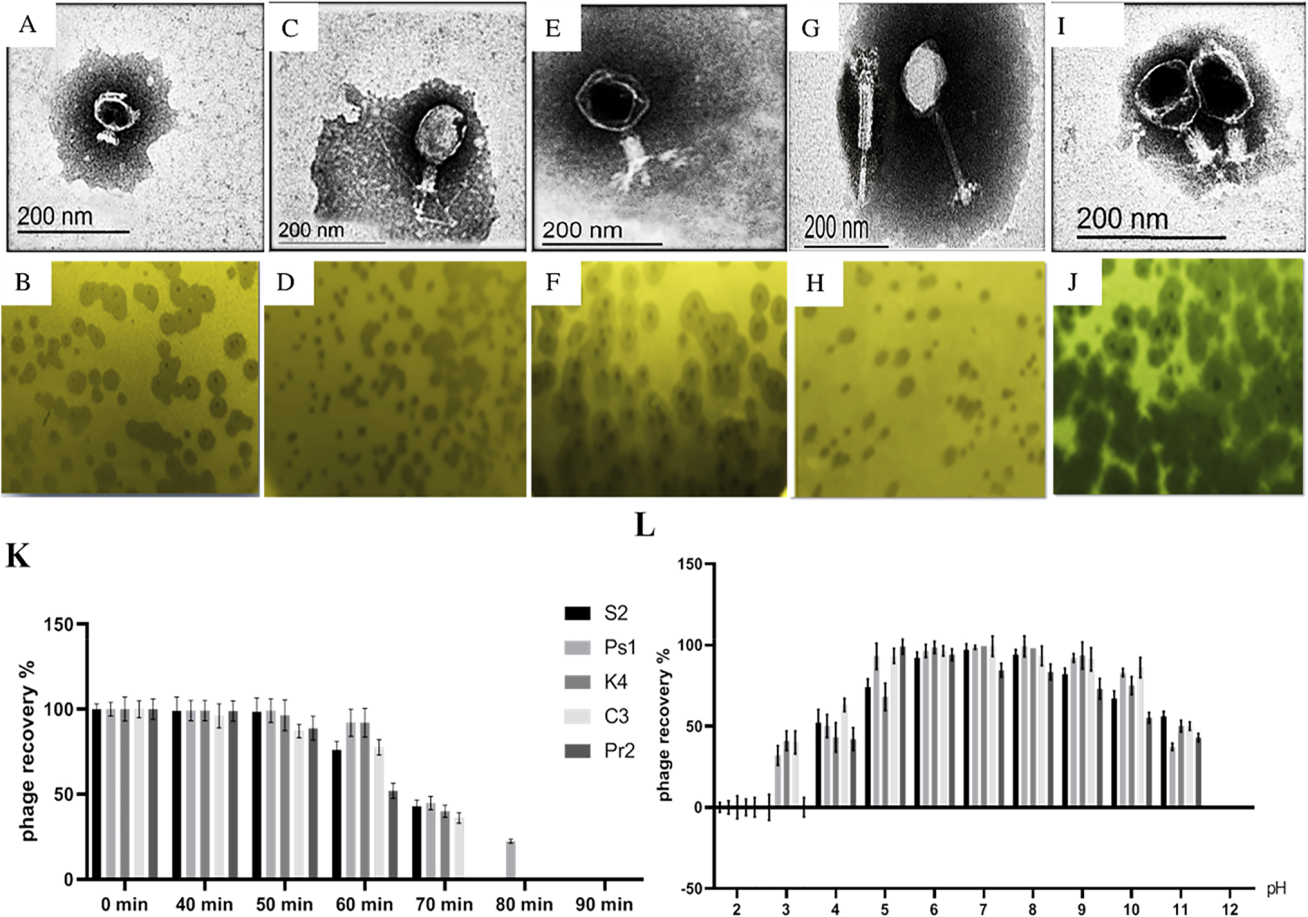
Transmission electron microscopy morphological characterization (**A**, **C**, **E**, **G**, **I**), plaque morphology for S2, Ps1, K4, C3, and Pr2 phages (**B**, **D**, **F**, **H**, **J**), thermal inactivation point for S2, Ps1, K4, C3, and Pr2 phage isolates (**K**), and stability of S2, Ps1, K4, C3, and Pr2 phages under different pH values (**L**)

Specific five phages for the selected multidrug-resistant *S. aureus* (LC596095), *P. aeruginosa* (LC586427), *K. variicola* (LC589614), *P. mirabilis* (LC589616), and *E. coli* (LC589615) were detected in clarified suspensions that were prepared from collected sewage samples using spot test method. These phages were confirmed by plaque assay and purified, then characterized according to plaque morphology and designated as S2 for *S*. *aureus* phage, Ps1 for *P. aeruginosa* phage, K4 for *K. variicola* phage, C3 for *E*. *coli* phage, and Pr2 for *P*. *mirabilis* phage. Transmission electron microscopy was used to classify the purified phages based on their virion morphology. The results revealed that the five phage particles each had a head and tail. The results showed that S2 phage belongs to the Podoviridae family and contains an isometric head with a diameter of 56.4 nm and a short tail with a length of 22.6 nm and a diameter of 19.04 nm, while Ps1 phage contains a cubic head with a diameter of 111.2 nm and a long contractile tail with a length of 109.6 nm and a diameter of 29 nm that terminates with tail fibers. Phage K4 specific for *K. variicola* contains a hexagonal head separated by a constricted neck region with a diameter of 112.9 nm and a tail with a length of 96.8 nm and a diameter of 29 nm. Moreover, C3 phage contains a hexagonal isometric head with a diameter of 88.7 nm and a long tail with a length of 80.6 nm with a diameter of 25.8 nm. Furthermore, Pr2 phage contains a hexagonal head with a diameter of 85.5 nm, separated from the head by a constricted neck region, and a long tail with a length of 151.6 nm and a diameter of 16.1 nm. From the results, Ps1, K4, C3, and Pr2 phages are assumed to belong to the Myoviridae family (Fig. 1A, C, E, G, I). All the aforementioned phages exhibited marked lytic activity Ps1 and Pr2 bacteriophages produced clear lytic plaques ranging from 2 to 4 mm in diameter. Plaques produced by the bacteriophages S2, K4, and C3 were surrounded by growing opaque halo zones (Fig. 1B, D, F, H, J), which also resisted the extremes of pH and temperature (Fig. 1K, L).

### Phage therapy in animal model

#### Phage toxicity test in rats

The results showed that the mean rectal temperature of all phage cocktail-injected rats was 36.9 °C compared to the temperature of the control group, which was 37.1 °C. No symptoms of irritation, lethargy, sickness, or fever were noted in the tested group during the period of observation. So, the phage cocktail showed no toxicity in rats and was considered for further use in vivo.

#### Validation of type 1 diabetes and diabetic wound onset

**Fig. 2:**
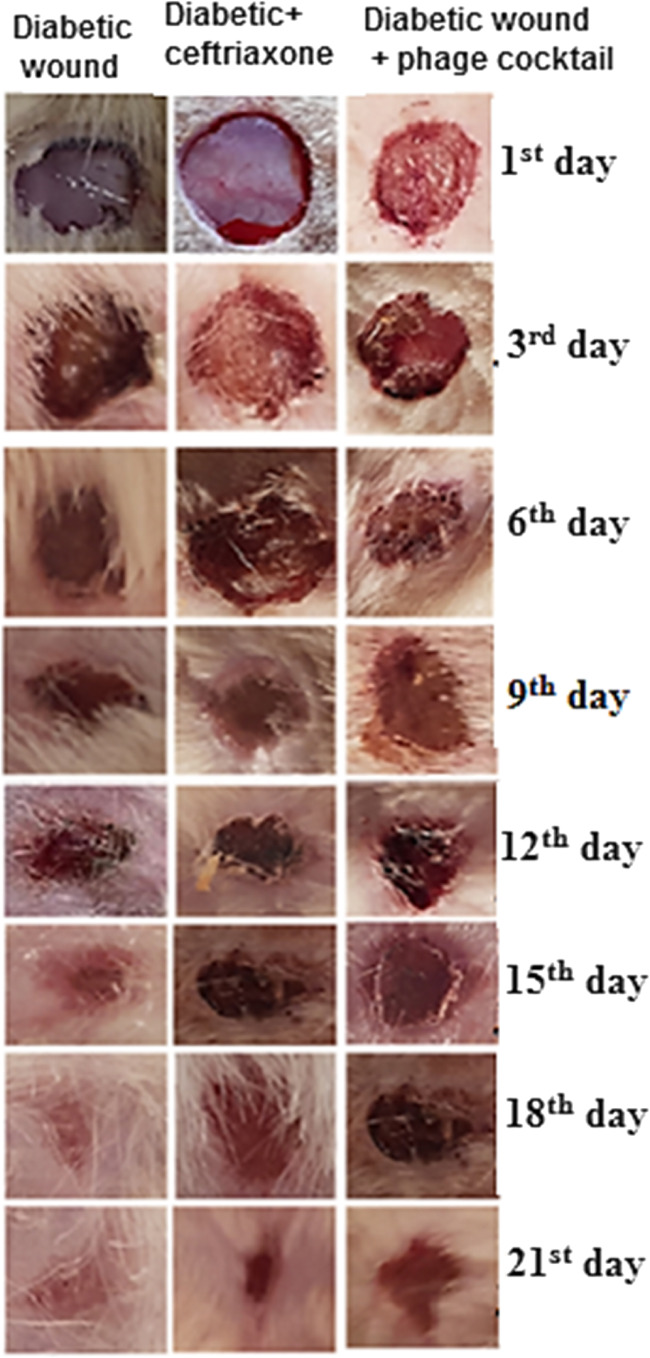
Wound imaging follow-up wound diameter on the 1st, 3rd, 6th, 9th, 12th, 15th, 18th, and 21st days post treatment. Values are mean of 8 rats per group ± SEM. Means bearing stars were significantly different at *P* < 0.05

The results of the present investigation illustrated that the streptozotocin (STZ) intraperitoneal injected rats developed both a sustained elevation in blood glucose level ≥ 100 mg/dl and a sharp decrease in serum insulin level < 1.1 mIU/ml as shown in Sup. 4A, B, with a progressive decrease in body weight within the first 1 week of diabetic induction. Three days post diabetic wound incision and infection with a cocktail of multidrug-resistant bacteria, the wound showed signs of suppuration and local bacterial infection without development of any signs of systemic bacterial infection (Fig. 2).

#### Effect of phage cocktail on wound healing

It was demonstrated that topical application of both the phage cocktail-treated group and the ceftriaxone-treated group elicited a significant reduction in the mean value (*P* < 0.0002 and *P* = 0.0002 of wound size) and wound index (*P* = 0.0002 and *P* = 0.007), respectively, compared to untreated diabetic rats, as shown in Sup. 4C, D and Fig. 2.

#### Wound microbial load

Bacterial load was assessed in terms of CFU/ml after 7 days in diabetic rats treated with phage cocktail and diabetic rats treated with antibiotics compared with untreated infected diabetic rats on nutrient and MacConkey agar plates. The obtained results showed that the bacterial load was increased in untreated infected animals since the bacterial count was 10.5 log and 9.5 log CFU/ml in nutrient and MacConkey agar, respectively, while in the group that received ceftriaxone topically, the bacterial load was significantly reduced to 7.5 and 5.5 log CFU/ml in nutrient and MacConkey agar, respectively. A significant reduction in bacterial load was obtained after phage cocktail 10^9^ PFU/ml treatments since a bacterial count of 4.5 log and 2.6 log CFU/ml in nutrient and MacConkey agar, respectively, as shown in Table 2. The values are the mean of triplicated bacterial counts ± SEM.

**Table 2:** Effect of the hydrogel topical application of ceftriaxone (2%) and phage cocktail (10^9^ PFU/g) at a multiplicity of infection (MOI = 10) once daily for 7 successive days on the mean value of total bacterial count CFU/ml (log_10_) of the diabetic wound infected with clinical isolates in type 1 diabetic rats on 21st days post-treatment

	Groups		
Media	Diabetic	Diabetic ceftriaxone treated	Diabetic phage cocktail-treated
Nutrient agar	10.5 ± 1.15^a^	7.5 ± 0.65^b^	4.5 ± 0.34^c^
MacConkey agar	9.5 ± 0.87^a^	5.5 ± 0.38^b^	2.6 ± 0.17^c^

^a,b,c^*P* < 0.05

#### Effect of phage cocktail on the expression of wound oxidative state

**Fig. 3:**
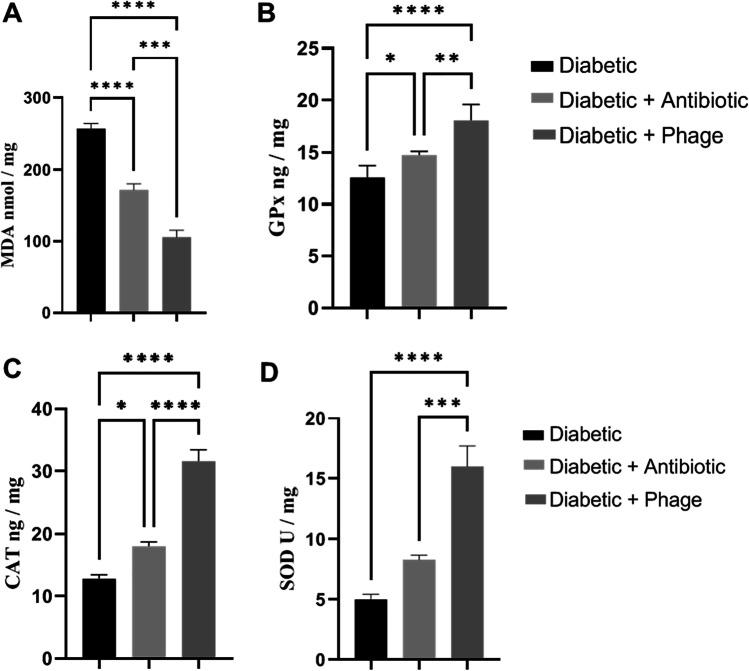
Effect of 2% ceftriaxone (µg/ml) and phage cocktail (10^9^ PFU/ml) on the mean value of oxidant/antioxidant activity of the type 1 diabetic rat wound (**A**, **D**). **A** Lipid peroxidation marker (MDA nmol/mg). **B** GPx ng/ mg. **C** CAT ng/mg. **D** SOD U/mg. Values are mean of 8 rats per group ± SEM. *Scant difference, **moderate difference, and ***distinctive difference

The results cited in Fig. 3A, D showed that both phage cocktail and ceftriaxone caused a significant decrease (*P* < 0.001) in the mean value of the lipid peroxidation marker MDA and a significant increase (*P* < 0.001) in the mean value of the diabetic wound antioxidant activity GPx and CAT ng/mg. On the other hand, only phage therapy elicited a significant increase (*P* < 0.001) in the mean value of the diabetic wound antioxidant activity SOD when compared with the ceftriaxone-treated group (*P* < 0.05).

#### Effect of phage cocktail and ceftriaxone on the expression of wound healing markers and collagen deposition

**Fig. 4:**
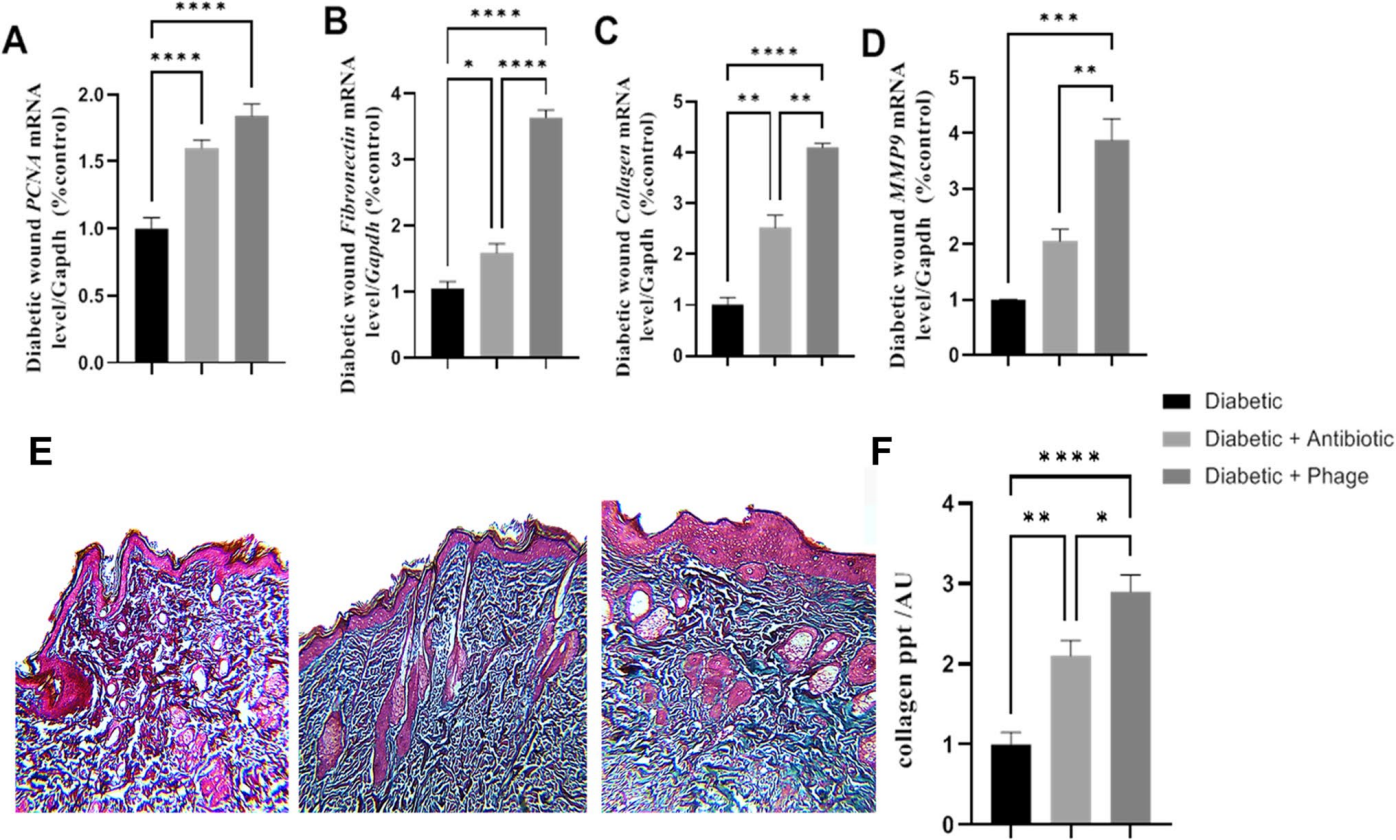
Effect of the hydrogel topical application of ceftriaxone 10% and phage cocktail 10.^9^ PFU/g at a multiplicity of infection (MOI = 10) once daily for 7 successive days on the mean fold change *mRNA* relative expression of regenerative markers (*PCNA*, *Fibronectin*, *COL1*, and *MMP9*) to internal control gene *Gapdh* of the diabetic wound infected with clinical isolates of *Staphylococcus aureus* LC596095, *Pseudomonas aeruginosa* LC586427, *Klebsiella variicola* LC589614, *Escherichia coli* LC589615, and *Proteus mirabilis* LC589616 in type 1 diabetic rats (**A**, **D**) and collagen deposition of the type 1 diabetic rats (**E**) of the type 1 diabetic rat wound. **A** mRNA relative expression of *PCNA* to internal control gene *Gapdh*. **B** mRNA relative expression of *Fibronectin* to internal control gene *Gapdh*. **C** mRNA relative expression of *COL1* to internal control gene *Gapdh*. **D** mRNA relative expression of *MMP9* to internal control gene *Gapdh*. **E** A photo micrograph of Masson’s blue staining of the control group (left panel), ceftriaxone-treated group (middle panel), and phage cocktail-treated group (right panel). **F** The level of collagen deposition. Values are mean of 8 rats per group ± SEM. Means bearing asterisk were significantly different at *P* < 0.05

The results of the present investigation indicated that either phage cocktail or antibiotic topical application induced a significant upregulation in the mean fold change of the mRNA expression of the healing regenerative biomarkers (*P* < 0.001) *PCNA*, *Collagen1*, *MMP9*, and collagen deposition compared with the untreated diabetic group. However, the phage cocktail-treated group showed a significant upregulation in the mean fold change of the mRNA expression of *Fibronectin* (*P* < 0.001) compared with the infected diabetic group or the ceftriaxone-treated one. On the other hand, the topical application of phage cocktail showed a significant *P* < 0.001 upregulation in the mean fold exchange of mRNA expression of *Collagen1*, *MMP9*, and collagen deposition compared with the ceftriaxone-treated diabetic group as shown in Fig. 4A, H.

#### Effect of phage cocktail on the expression of wound inflammatory and anti-inflammatory markers

**Fig. 5:**
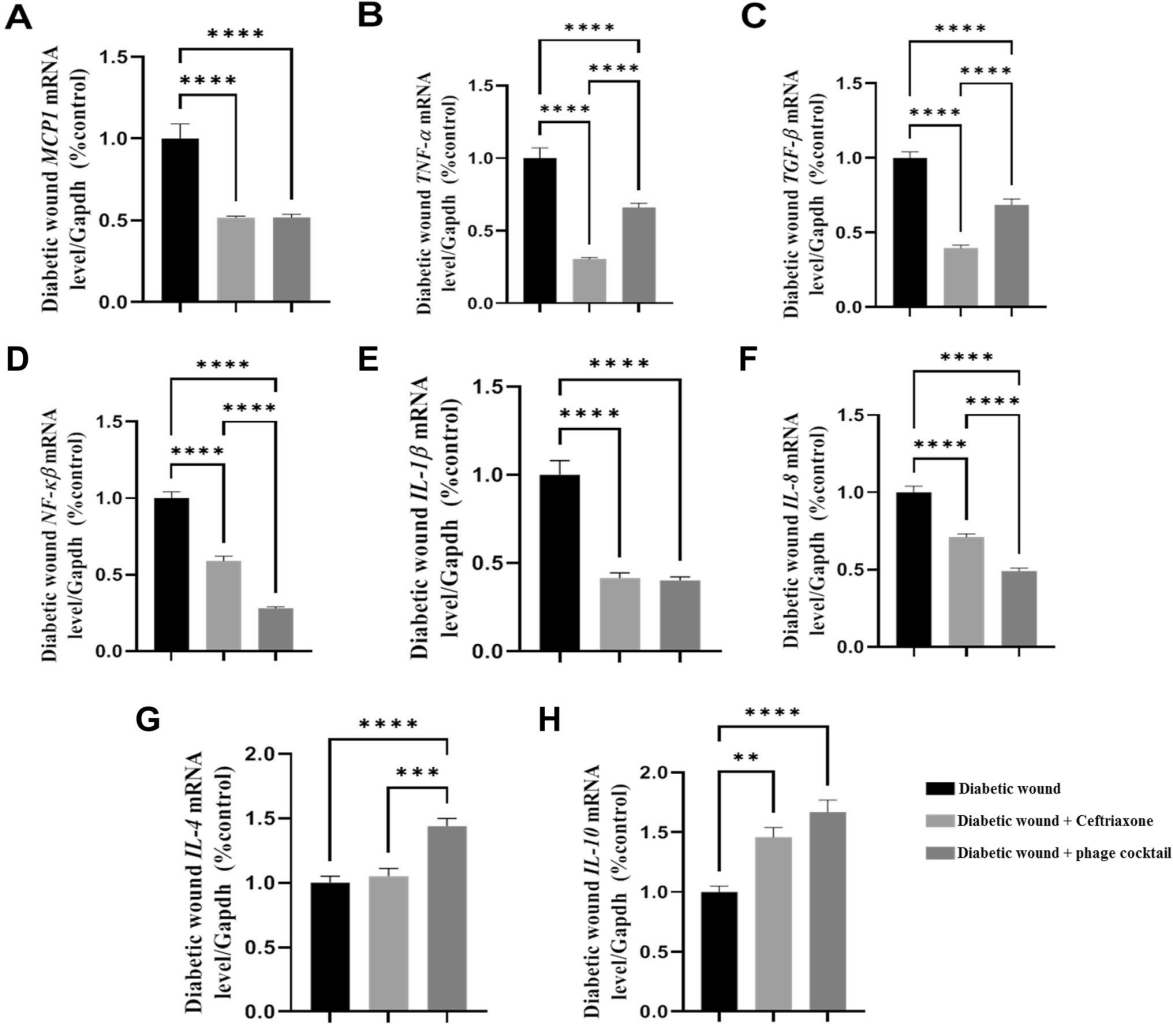
Effect of the hydrogel topical application of ceftriaxone 10% and phage cocktail 10^9^ PFU/g at a multiplicity of infection (MOI = 10) once daily for 7 successive days on the mean fold change of the *mRNA* relative expression of inflammatory and anti-inflammatory markers to internal control gene *Gapdh* of the diabetic wound infected with clinical isolates of *Staphylococcus aureus* LC596095, *Pseudomonas aeruginosa* LC586427, *Klebsiella variicola* LC589614, *Escherichia coli* LC589615, and *Proteus mirabilis* LC589616 in type 1 diabetic rats (**A**, **H**). **A** mRNA relative expression of *MCP1* to internal control gene *Gapdh*. **B** mRNA relative expression of *TNF-α* to internal control gene *Gapdh*. **C** mRNA relative expression of *TGF-β* to internal control gene *Gapdh*. **D** mRNA relative expression of *NF-κβ* to internal control gene *Gapdh*. **E** mRNA relative expression of *IL-1β* to internal control gene *Gapdh*. **F** mRNA relative expression of *IL-8* to internal control gene *Gapdh*. **G** mRNA relative expression of *IL-4* to internal control gene *Gapdh*. **H** mRNA relative expression of *IL-10* to internal control gene *Gapdh*. **Scant difference, ***moderate difference, and ****distinctive difference

The results of the present study illustrated that either phage cocktail or antibiotic topical application induced a significant downregulation in the mean fold change of the mRNA expression of the inflammatory biomarkers (*P* < 0.001) *MCP1*, *TNF-α*, *TGF-β*, *NFK-β*, *IL-1β*, and *IL-8* compared to the untreated infected diabetic group. Moreover, phage therapy showed a significant *P* < 0.001 improvement in the mRNA expression of the aforementioned inflammatory markers except *TNF-α* and *TGF-β* and anti-inflammatory markers over the antibiotic one. On the same line, antibiotic therapy evoked a significantly downregulated mRNA expression of TNF-α and TGF-β over compared to phage therapy. In contrast, only phage therapy elicited a significant upregulation *P* < 0.001 in the mean fold exchange of the anti-inflammatory marker *IL-4* in comparison to the infected diabetic group, while both phage cocktail and antibiotic topical therapy elicited a significant upregulation in the mean fold exchange of the anti-inflammatory marker *IL-10* in comparison to the infected diabetic group (Fig. 5A, F).

#### Effect of phage cocktail and ceftriaxone on the histopathological picture of type 1 diabetic wound

**Fig. 6:**
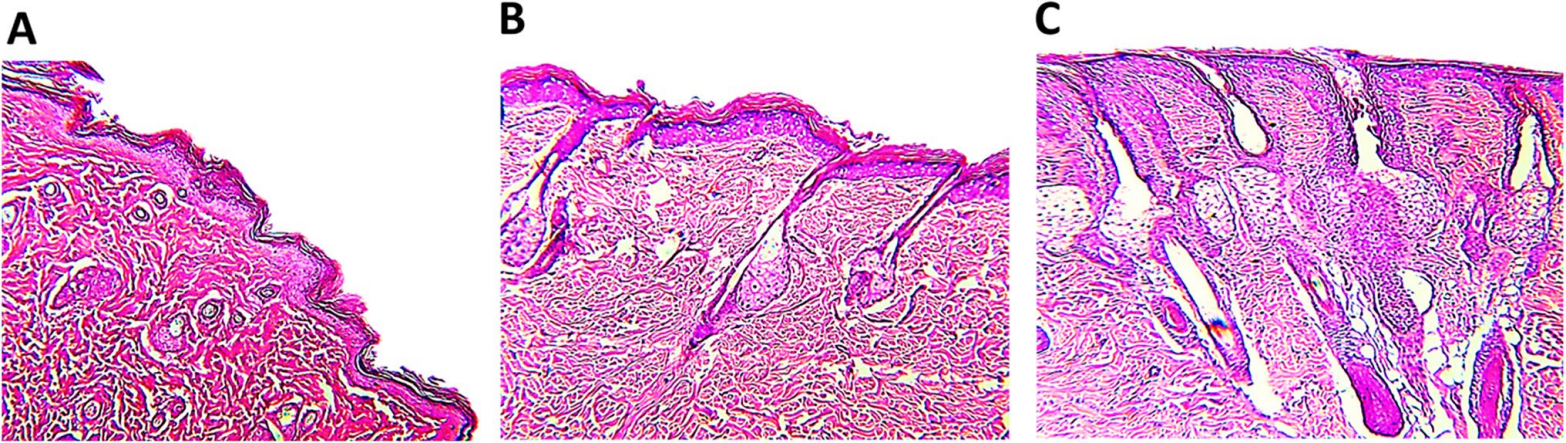
Effect of the topical application of phage cocktail on diabetic infected wound in type 1 diabetic rats (**A**, **C**). **A** A photomicrograph of infected diabetic wound H&E, × 400. **B** A photomicrograph of diabetic infected wound cefetriaxone-treated group H&E, × 400. **C** A photomicrograph of diabetic infected wound phage cocktail-treated group H&E, × 400

Histopathological examination of the hemotoxylin (H) and eosin (E) stained section of the non-treated diabetic wound showed signs of acute inflammation, congested blood vessels, massive leukocytic infiltration, and the appearance of vascular granulation tissue as shown in Fig. 6A. However, the histomorphic picture of the ceftriaxone-treated diabetic wound revealed moderate leukocytic infiltration with the formation of epidermal pads, the appearance of hair follicles, and collagen that indicated the common signs of wound healing as shown in Fig. 6B. On the other hand, histomorphological examination of phage cocktail-treated diabetic wound illustrated a mild inflammatory reaction, appearance of moderate number of fibroblast along with granulation tissue, development of epidermal pads, increasing epidermal thickness, and restoration of both hair follicles and sebaceous gland that indicated an advanced regenerative state of the damaged diabetic skin as shown in Fig. 6C.

## Discussion

Diabetic foot ulcers are one of the most serious complications of diabetes that deteriorate the patient’s quality of life and are considered suitable media for the growth and multiplication of the invading pathogens due to the retardation in wound healing with prolonged regimens of both systemic and/or topical antimicrobial therapy. Multidrug-resistant pathogens have a sharp need for safe and effective antimicrobial alternatives. Thus, the present study was designed to investigate whether phage therapy could be considered as an alternative strategy to the antimicrobial one.

In the present study, it was found that the proportion of Gram-negative bacilli was higher than Gram-positive cocci. These results were compatible with Shanmugam et al. (2013). From 78 positive culture cases, 10 patients (12.8%) had monomicrobial infections and 68 patients (87.2%) had polymicrobial infections. Similarly, Citron et al. (2007) reported that 16.2% of patients had monomicrobial infections and 83% had polymicrobial infections. In the same line, Miyan et al. (2017) showed that 56.87% of the DFIs were polymicrobial with a high abundance of Gram-negative isolates (76.27%). In this study, the commonest isolates were *Staphylococcus aureus* (24%), followed by *Pseudomonas aeruginosa* (18.2%), *Klebsiella* spp. (17.3%), *Proteus mirabilis* (15.6%), *Escherichia coli* (12.9%), *Acinetobacter baumannii* (4.8%), *Enterococcus faecalis* (4.8%), and *Enterobacter cloacae* (2.4%). Similarly, Saltoglua et al. (2018) reported that the most common isolated microorganisms from diabetic foot ulcers were *S*. *aureus* (20%), *P*. *aeruginosa* (19%), and *E*. coli (12%). On the other hand, Xavier et al. (2014) reported that the predominant bacteria isolated from wounds were *E. coli* (16.3%), *P. aeruginosa* (10.7%), *E. faecalis* (9.2%), *Klebsiella pneumoniae* (8.9%), *S. aureus* (8.9%), *Acinetobacter baumannii* (5.1%), *Enterobacter* sp. (4.9%), and *Proteus mirabilis* (3.3%). Also, Shanmugam et al. (2013) reported that Gram-negative bacilli were more prevalent (65.1%) than Gram-positive cocci (39.8%). The commonest isolate was *Pseudomonas* sp. (16%), followed by *Escherichia coli* (14.6%) and *Staphylococcus aureus* (13.3%). The diabetic foot microbiota has been found to be influenced by several factors such as demographic characteristics, personal hygiene, geographical origin of the patient, grade of severity, glycemic control, and ongoing or previous antibiotic treatments (Spichler et al. 2015; Malone et al. 2017).

The antibiotic resistance patterns of the isolated Gram-negative bacilli and the Gram-positive cocci were studied using twenty-five different antibiotics. It was found that the isolates were multidrug resistant with percentages of 80%, 73.7%, 83.3%, 68.75%, 92%, 80%, 90%, and 100% for *Staphylococcus* sp., *P. aeruginosa*, *Klebsiella* sp., *Proteus* sp., *E*. *coli*, *Acinetobacter* sp., *Enterococcus* sp., and *Enterobacter* sp., respectively.

To examine the therapeutic effect of bacteriophages in resolving diabetic foot infection, five phages specific for *S*. *aureus*, *P*. *aeruginosa*, *K*. *variicola*, *E*. *coli*, and *P*. *mirabilis* were isolated from two sewage samples obtained from different sources, which formed distinct plaques which differ in size and transparency. S2, K4, and C3 bacteriophages produced clear lytic plaques ranging from 3 to 4 mm in diameter and surrounded by growing opaque halo zones, while Ps1 and Pr2 phages produced clear lytic plaques with a diameter of 2 mm without halo. The formation of halo is often interpreted as an indicator of depolymerase-mediated digestion of bacterial capsules in nearby bacteria even if lysis does not occur in these areas (Shang et al. 2015; Pan et al. 2017).

According to ICTV, S2 phage belongs to the family Podoviridae and contains an isometric head and a short non-contractile tail. These results are in agreement with Son et al. (2010) and Cha et al. (2019), who isolated two phages specific for *S*. *aureu*s, which possesses a small icosahedral head with a short, non-flexible, non-contractile tail that belongs to the Podoviridae family. The morphological characteristics of *Pseudomonas* phage in the current study revealed that Ps1 contains a cubic head and a long contractile tail terminating in tail fibers and belongs to the Myoviridae family. This data is in agreement with Guo et al. (2019), Garbe et al. (2011), and Angela et al. (2015). In contrast, Shigehisa et al. (2016) isolated phage KPP21 that belongs to the family Podoviridae with a head diameter and tail length of 67.0 nm and 5.3 nm, respectively. Transmission electron microscopy also revealed that the K4 phage belongs to the Myoviridae family. In the same regard, Hesse et al. (2020) isolated P6 phage from sewage samples that belong to Myoviridae. On the other hand, Teng et al. (2019) and Domingo-Calap et al. (2020) isolated *Klebsiella* phages that possess an isometric head and a non-contractile short tail resembling those of Podoviridae family members. The C3 phage belongs to the Myoviridae family. These results are compatible with Necel et al. (2020), who isolated two phages, mvB_Eco4M-7 and ECML-117, that are specific for *E. coli* O157:H7 (ST2–8624) and belong to the Myoviridae family. These phages appear in electron microscopic analyses with a head of 66 nm in diameter for both phages and a contractile tail with a diameter of 107 × 20 nm and 120 × 20 nm for vB_Eco4M-7 and ECML-117, respectively. In addition, Pr2 phage contains a hexagonal head with a diameter of 85.5 nm, separated from the head by a constricted neck region and a long tail with a length of 151.6 nm with a diameter of 16.1 nm and belongs to the Myoviridae family. These data are similar to Luís et al. (2016) who isolated two phages specific for *P. mirabilis* from raw sewage which formed clear plaques with diameter ranged between 1 and 1.5 nm and contain isometric head 87 nm in diameter and contractile tail (110 nm long and 17 nm wide) and belongs to the Myoviridae family but the other phage 5460 had a capsid 65 nm in diameter, short (13 nm) tail terminating in tail fibers, suggesting that these probably belong to the family Podoviridae.

Upon investigation of using bacteriophage therapy as an effective alternative for the antibiotic therapeutic regimens for the polymicrobial DFU, the results of the present study revealed that diabetic rats showed sustained elevation in blood glucose level ≥ 500 mg/dl and sharp decrease in serum insulin level as < 3.5 mIU/ml with a progressive decrease in body weight within the first 3 weeks of diabetic induction. These results are compatible with Molehin and Oloyede (2019) and Kifelew et al. (2020).

The current study demonstrated the potential effectiveness of topical administration of specific phage cocktails on survival and bacterial loads in a mouse model of mixed infected diabetic wounds compared with topical administration of ceftriaxone treatment. The results revealed that the topical administration of both phage cocktail and ceftriaxone showed a significant decrease in wound size and wound index compared to untreated diabetic rats. These results are in agreement with Chhibber et al. (2013) and Kifelew et al. (2020).

Lipid peroxidation by-products can result in marked damage to the functional and structural integrity of cell membranes (Kolanjiappan et al. 2002). Antioxidants such as GSH, SOD, CAT, and GPx can remove the by-products of lipid peroxidation (Manoharan et al. 2005). An imbalance between the antioxidants and lipid peroxidation by-products can lead to cell and tissue damage (Nagini and Saroja 2001). The current work showed that both phage cocktail and ceftriaxone treatments caused a significant decrease *P* < 0.001 in the mean value of the lipid peroxidation marker MDA and a significant increase *P* < 0.001 and *P* < 0.05, respectively, in the mean value of the antioxidant enzyme activity GPx and CAT ng/mg. On the other hand, phage therapy elicited a significant increase *P* < 0.001 in the mean value of the diabetic wound antioxidant activity, either GPx or SOD or CAT, compared with the ceftriaxone-treated group. In the same respect, Mahreen et al. (2010) and Rani et al. (2019) reported that there was a significant increase in the malondialdehyde (MDA) level in noncomplicated and complicated diabetics with nephropathy as compared to healthy controls. Moreover, Chhibber et al. (2013) estimated the antioxidant myeloperoxidase (MPO) activity and found a significant reduction in the MPO activity in all treated groups with the phage MR-10 group and linezolid group as compared to untreated control animals (*P* < 0.01) with minimal activity obtained on days 7 and 10.

The results also showed that the topical application of the phage cocktail caused a marked reduction in the wound microbial load and also showed a superior effect over the ceftriaxone-treated group. These results are in accordance with Kifelew et al. (2020). In the same line, Chhibber et al. (2013) reported that both the phage MR-10-treated group and the linezolid (orally)-treated group showed a significant reduction of 2.7 and 2.92 log CFU, respectively, as compared to normal animals on day 5. Our finding could be attributed to the ability of the phage to replicate at the site of infection, infect the bacterial cells, and produce several lytic enzymes that destroy bacterial cells for an extended period. In contrast to the antibiotic candidates, phage showed no local inflammatory reaction, provided the flexibility to introduce higher PFU with a small dose, and had higher potency to replicate in the site of the infection that extended the therapeutic effect with minimum or reduced dosage regimens compared to the antibiotics, and the topical application of the phages reduced the risk of both first path metabolism and systemic side effects (Abbas et al. 2015; Speck and Smithyman 2016).

Wound healing process was associated with the expression of several proteins as collagen (Coli-1), fibronectin (Fn), matrix metalloproteinase 9 (MMP9), and matrix metalloproteinase 3 (MMP3) (Suvik and Effendy 2012). The abovementioned proteins are essential for proper wound healing. However, diabetes disrupts the expression of these proteins, which negatively impacts diabetic wound healing (Loots et al. 1998; Pollack et al. 2016), since these proteins are responsible for extracellular matrix deposition and tissue remodelling. So the dysregulation of their expression limits the progression of the wound remodelling process (Shi et al. 2015).

Results of regenerative markers in this study revealed that the topical application of phage cocktail showed a significant *P* < 0.001 upregulation in the mean fold exchange of mRNA expression of *Collagen1*, *MMP9*, and collagen deposition compared with the ceftriaxone-treated diabetic group. Furthermore, the phage cocktail-treated group showed a significant upregulation in the mean fold change of the mRNA expression of *Fibronectin P* < 0.001 compared with the ceftriaxone-treated group. The untreated diabetic group showed a highly ulcerated epidermal layer and pus-containing abscesses in both the dermis and subcutaneous soft tissue. The topical application of a phage cocktail appeared to be the first option for inducing the expression of the wound healing proteins over the antibiotic one (Zhang et al. 2014; Abbas et al. 2015). The lower therapeutic capacity of the antibiotics than the phage might be illustrated as follows: the improper use of antibiotics can lead to an increase in bacterial resistance (Zaman et al. 2017). Chemotherapeutic agents commonly cause direct toxic effects such as rashes that adversely affect wound healing; antibiotics usually disrupt the skin flora that eventually impair many aspects of the innate immunity, thereby interfering with the wound healing process; and antibiotics can interact with other medications to cause drug-related problems (Zhang et al. 2014). The obtained results illustrated that the topical application of either phage cocktail or antibiotics induced a significant downregulation in the mean fold change of the mRNA expression of the inflammatory biomarkers (*P* < 0.001) *MCP1*, *TNF-α*, *TGF-β*, *NFKβ*, *IL-1β*, and *IL-8* compared to the diabetic group. In contrast, only the phage therapy elicited a significant upregulation *P* < 0.001 in the mean fold exchange of the anti-inflammatory marker *IL-4* in comparison to the diabetic group. Moreover, phage therapy showed a significant *P* < 0.001 improvement in the mRNA expression of the forementioned inflammatory marker except (*TNF-α* and *TGF-β*) and anti-inflammatory markers over the antibiotic one. In the same line, antibiotic therapy evoked a significantly downregulated mRNA expression of TNF-α and TGF-β over compared to phage therapy. These results were correlated with those of Kumari et al. (2010). In the same line, Pincus et al. (2015) reported that SATA-8505 phage did not induce inflammatory responses either to IL-1ß or IL-6 in peripheral blood mononuclear cultures, nor did it induce interferon gamma production in primary human keratinocyte cultures or induce inflammatory responses in mouse models. In the same regard, Berlina et al. (2020) mentioned that the main factors determining the levels of inflammatory markers in the blood of diabetic patients were as follows: the severity of the disease and its duration; the etiology of the disease (bacterial, fungal, or viral infections, superinfection mixed infections); administration of antiviral drugs or antibiotics; and the stage of antibiotic therapy (initial, final).

The results of histopathological examination of the H&E-stained section of the non-treated diabetic wound showed signs of acute inflammation, congested blood vessels, massive leukocytic infiltration, and the appearance of vascular granulation tissue. However, the histomorphic picture of the ceftriaxone-treated diabetic wound revealed moderate leukocytic infiltration with the formation of epidermal pads, the appearance of hair follicles, and collagen that indicated the common signs of wound healing. On the other hand, histomorphological examination of phage cocktail-treated diabetic wounds illustrated a mild inflammatory reaction, the appearance of a moderate number of fibroblasts along with granulation tissue, the development of epidermal pads, increasing epidermal thickness, and restoration of both hair follicles and sebaceous glands that indicated an advanced regenerative state of the damaged diabetic skin. These results are similar to those of Chhibber et al. (2013) and Chhibber et al. (2018), who found that the skin of both the phage-treated group and the linezolid-treated group showed mild infiltration of lymphocytes and fibroblastic cells in the dermis beneath the epidermis. Although the epidermis appeared normal and non-ulcerated, moderate inflammation was observed in the dermis beneath the epidermis, with edema fluid also visible in the dermal layer. On the other hand, the non-diabetic skin showed all the intact layers (epidermis with keratin layers), dermis with hair follicles being visible, and the fat and muscle layer. The phage cocktail used in the current study was tolerated by the rats by the lack of changes in the well-being of rats or anaphylactic reactions due to harmful responses to phages. These findings are in the same line with studies on the safety of phage therapy (Fabijan et al. 2020; Kifelew et al. 2020). Adverse effects due to rapid bacterial lysis, such as circulatory shock and bacterial rebound due to releasing large quantities of toxins when considerable numbers of bacteria are lysed, or anti-inflammatory responses were not observed (Van Belleghem et al. 2017; Sweere et al. 2019). However, the use of phage therapy can be an effective treatment against bacterial infections; there are some important factors that considered a challenge to the use of phage therapy as antimicrobial agent. One drawback is the use of phage therapy against intracellular pathogens (Sulakvelidze et al. 2001). Moreover, major concerns were about the potentially massive endotoxins that liberated after bacterial lysis (Mignon et al. 2014). So, this is an area to monitor in phage therapy clinical trials and more detailed studies are necessary. So, the proper manipulation of these highly active phages, full genome sequencing, and protein analysis can be the ultimate keys for better application.

## Conclusion

Diabetic foot is one of the most feared complications of diabetes and is characterized by several pathological complications such as foot ulceration, which frequently becomes infected with bacteria. Healing of these ulcers is largely delayed by the concomitant infection. The looming threat of antibiotic resistance calls for immediate action. Phage therapy is well suited to be part of the multidimensional strategies to fight against it. The results of this study suggested topical phage therapy was a promising method for the treatment of non-healing diabetic wounds and did not exhibit any side effects, ensuring their safety. So, it was recommended to increase the application scale of phage therapy to include diabetic foot patients. The proper manipulation of these highly active phages, full genome sequencing, and protein analysis can be the ultimate keys for better application.

## Sample availability

Samples of the compounds are not available from the authors.

## Supplementary Information

Below is the link to the electronic supplementary material.

Supplementary file1 (DOCX 928 KB)

## Data Availability

The data that support the findings of this study are available from the corresponding author upon reasonable request.

## References

[CR1] Abbas M, Uc KI, Lipsky BA (2015). In diabetic foot infections antibiotics are to treat infection, not to heal wounds. Exper Opin Pharmaco.

[CR2] Adams MH (1959). Bacteriophages interscience Publishers Inc. N Y.

[CR3] Akhi MT, Shirinzadeh M, Ghotaslou R, Sorous MH, Pirzadeh T, Behzad MN (2013). Determination of antibiotic sensitivity of *Bacteroid fragilis* isolated from patients and healthy individuals in Imam Reza Center of Medical Teaching and Treatment Tabriz. Jund J Microbiol.

[CR4] Altschul SF, Gish W, Miller W, Myers EW, Lipmanl DJ (1990). Basic local alignment search tool. J Mol Biol.

[CR5] Angela VH, Rangel G, Clavijo V, Prada C, Mantilla M, Gomez MC, Kutter E, Taylor C, Fineran PC, Barrios AFG, Vives MJ (2015). Phage _Pan70, a putative temperate phage, controls *Pseudomonas aeruginosa* in planktonic, biofilm and burn mouse model assays. Viruses.

[CR6] Bancroft, JD Layton C (2019) Bancroft’s theory and practice of histological techniques E-Book. In: Kim Suvarna S, Layton C, Bancroft JD (eds) Elsevier Health Sciences, pp 126–137

[CR7] Bauer A, Kirby W, Sherris J, Turck M (1966). Antibiotic susceptibility testing by a standardization single disk method. Am J Clin Pathol.

[CR8] Berlina AN, Zherdey AV, Dzantiev BB (2020). Monitoring antibiotics and inflammatory markers in human blood: impact in choice of antibiotic therapy and used methods. Biomed Pharm J.

[CR9] Bolocan AS, Callanan J, Forde A, Ross P, Hill C (2016). Phage therapy targeting *Escherichia coli* a story with no end?. FEMS Microbiol Lett.

[CR10] Bradley DE (1967). Ultrastructure of bacteriophage and bacteriocins. Bacteriol Rev.

[CR11] Butler MS, Blaskovich MAT, Cooper MA (2017). Antibiotics in the clinical pipeline at the end of 2015. J Antibiotic.

[CR12] Castillo DE, Nanda S, Keri JE (2019). *Propionibacterium* (Cutibacterium) acnes bacteriophage therapy in acne: current evidence and future perspectives. Dermatol Therapy.

[CR13] Centers for Disease Control and Prevention (CDC) (2013) National diabetes fact. Estimates of diabetes and its burden in the united states

[CR14] Cha K, Oh HK, Jang JY, Jo Y, Kim WK, Ha GU, Ko KS, Myung H (2018). Characterization of two novel bacteriophages infecting multidrug-resistant (MDR) *Acinetobacter baumannii* and evaluation of their therapeutic efficacy *in vivo*. Front Microbiol.

[CR15] Cha Y, Chun J, Son B, Ryu S (2019). Characterization and genome analysis of *Staphylococcus aureus* Podovirus CSA13 and its anti-biofilm capacity. Viruses.

[CR16] Chhibber S, Kaur T, Kaur S (2013). Co-therapy using lytic bacteriophage and linezolid: effective treatment in eliminating methicillin resistant *Staphylococcus aureus* (MRSA) from diabetic foot infections. PLoS ONE.

[CR17] Chhibber S, Kaur J, Kaur S (2018). Liposome entrapment of bacteriophages improves wound healing in a diabetic mouse MRSA infection. Front Microbiol.

[CR18] Citron DM, Goldstein EJ, Merriam CV, Lipsky BA, Abramson MA (2007). Bacteriology of moderate-to-severe diabetic foot infections and *in vitro* activity of antimicrobial agents. J Clin Microbiol.

[CR19] Dhar B, Singh B, Singh R, Singh R, SinghV SJ (1978). Isolation of a virus (RL1) infective on *Rhizobium leguminosurum*. Arch Microbiol.

[CR20] Domingo-Calap P, Beamud B, González-Candelas LMF, Sanjuán R (2020). Isolation and characterization of two *Klebsiella pneumoniae* phages encoding divergent depolymerases. Int J Mol Sci.

[CR21] El-Telbany M, El-Didamony G, Askora A, Ariny E, Abdallah D, Connerton F, El-Shibiny A (2021). Bacteriophages to control multi-drug resistant *Enterococcus faecalis* infect Dent Root Canal. Microorgan.

[CR22] Fabijan AP, Lin RC, Ho J, Maddocks S, Zakour NLB, Iredell JR (2020). Safety of bacteriophage therapy in severe *Staphylococcus aureus* infection. Nat Microbiol.

[CR23] Funke G, Monnet D, Bernardis C, Graevenitz A, Freney J (1998). Evaluation of the VITEK2 system for rapid identification of medically relevant Gram–negative rods. J Clin Microbiol.

[CR24] Garbe J, Bunk B, Rohde M, Schobert M (2011). Sequencing and characterization of *Pseudomonas aeruginosa* phage JG004. BMC Microbiol.

[CR25] Guo Y, Chen P, Wang LZ, T,  (2019). Characterization of two *Pseudomonas aeruginosa* viruses vB_PaeM_SCUT-S1 and vB_PaeM_SCUT-S2. Viruses.

[CR26] Hesse S, Rajaure M, Wall E, Johnson J, Bliskovsky V, Gottesman S, Adhyaa S (2020). Phage resistance in multidrug-resistant *Klebsiella pneumonia* ST258 evolves via diverse mutations that culminate in impaired adsorption. Mbio.

[CR27] Khamis T, Abdelalim AF, Abdallah SH, Saeed AA, Edress NM, Arisha AH (2020). Early intervention with breast milk mesenchymal stem cells attenuates the development of diabetic-induced testicular dysfunction via hypothalamic Kisspeptin/Kiss1r-GnRH/GnIH system in male rats. Biochim Biophys Acta (BBA) Mol Basis Dis.

[CR28] Kifelew LG, Warner MS, Morales S, Vaughan L, Woodman R, Fitridge R, Mitchell JG, Speck P (2020). Efficacy of phage cocktail AB-SA01 therapy in diabetic mouse wound infections caused by multidrug-resistant *Staphylococcus aureus*. BMC Microbiol.

[CR29] King AJ (2012). The use of animal models in diabetes research. Brit J Pharmacol.

[CR30] Kolanjiappan K, Manoharan S, Kayalvizhi M (2002). Measurement of erythrocyte lipids, lipid peroxidation, antioxidants and osmotic fragility in cervical cancer patients. Clin Chim Acta.

[CR31] Krumperman PH (1983). Multiple antibiotic resistance indexing *Escherichia coli* to identify risk sources of faecal contamination of foods. Appl Environ Microbiol.

[CR32] Kumari S, Harjaim K, Chhibber S (2010). Evidence to support the therapeutic potential of bacteriophage Kpn5 in burn wound infection caused by *Klebsiella pneumoniae* in BALB/c mice. J Microbiol Biotechnol.

[CR33] Loots MA, Lamme EN, Zeegelaar J, Mekkes JR, Bos JD, Middelkoop E (1998). Differences in cellular infiltrate and extracellular matrix of chronic diabetic and venous ulcers versus acute wounds. J Invest Dermatol.

[CR34] Luís DR, Veiga P, Cerca N, Kropinski AM, Almeida C, Azeredo J, Sillankorva S (2016). Development of a phage cocktail to control *Proteus mirabilis* catheter-associated urinary tract infections. Front Microbiol.

[CR35] Maciejewska B, Olszak T, Drulis-Kawa Z (2018). Applications of bacteriophages versus phage enzymes to combat and cure bacterial infections: an ambitious and also a realistic application. Appl Microbiol Biotechnol.

[CR36] Mahgoub EM, Elfatih M, Omer A (2015). Aerobic bacteria isolated from diabetic septic wounds. Am J Res Comm.

[CR37] Mahreen R, Mohsin M, Nashreen Z, Siraj M, Ishaq M (2010). Significantly increased levels of serum malondialdehyde in type 2 diabetics with myocardial infarction. Int J Diabet Develop Count.

[CR38] Malone M, Gosbell IB, Dickson HG, Vickery K, Espedido BA, Jensen SO (2017). Can molecular DNA-based techniques unravel the truth about diabetic foot infection*?*. Diabetes/metabolism Res Rev.

[CR39] Manoharan S, Kolanjiappan K, Suresh K, Panjamurthy K (2005). Lipid peroxidation and antioxidants status in patients with oral squamous cell carcinoma. Ind J Med Res.

[CR40] Mignon F, Piagnerelli M, van Nuffelen M, Vincent JL (2014). Effect of empiric antibiotic treatment on plasma endotoxin activity in septic patients. Infection.

[CR41] Miyan Z, Fawwad A, Sabir R, Basit A (2017). Microbiological pattern of diabetic foot infections at a tertiary care center in a developing country. Age Year.

[CR42] Molehin OR, Oloyede OI (2019). Attenuation of oxidative stress and hepatic damage by white butterfly (*Clerodendrum volubile*) leaves in streptozotocin-induced diabetes in rats. J Basic Clin Physiol Pharmacol.

[CR43] Muhammad AA, Arulselvan P, Cheah PS, Abas F, Fakurazi S (2016). Evaluation of wound healing properties of bioactive aqueous fraction from *Moringa oleifera* Lam on experimentally induced diabetic animal model. Drug Design Develop Ther.

[CR44] Nagini S, Saroja M (2001). Circulating lipid peroxides and antioxidants as biomarkers of tumor burden in patients with oral squamous cell carcinoma. Int J Biochem Biophys Mol Biol.

[CR45] Necel A, Bloch A, Nejman-Faleńczyk S, Grabski B, Topka M, Dydecka G, Kosznik-Kwaśnicka K, Grabowski Ł, Jurczak-Kurek A, Wołkowicz T, Węgrzyn G, Węgrzyn A (2020). Characterization of a bacteriophage, vB_Eco4M-7, that effectively infects many *Escherichia coli* O157 strains. Scientific Report.

[CR46] Noor S, Zubair M, Ahmad J (2015). Diabetic foot ulcer – a review on pathophysiology, classification and microbial etiology. Diab Metab Syndr.

[CR47] Pallavali RR, Degati VL, Lomada D, Reddy MC, Durbaka VRP (2017). Isolation and *in vitro* evaluation of bacteriophages against MDR-bacterial isolates from septic wound infections. PLoS ONE.

[CR48] Pan YJ, Lin TL, Chen CC, Tsai YT, Cheng YH, Chen YY, Hsieh PF, Lin YT, Wang JT (2017). *Klebsiella* phage ΦK64-1 encodes multiple depolymerases for multiple host capsular types. J Virol.

[CR49] Park S, Rich J, Hanses F, Lee JC (2009). Defects in innate immunity predispose C57BL/6J-Leprdb/Leprdb mice to infection by *Staphylococcus aureus*. Inf Immun.

[CR50] Philipson L, Albertson P, Frick G (1960). Purification and concentration of virus by aqueous polymer phase system. Virol.

[CR51] Pincus NB, Reckhow JD, Saleem D, Jammeh ML, Datta SK, Myles IA (2015). Strain specific phage treatment for *Staphylococcus aureus* infection is influenced by host immunity and site of infection. PLoS ONE.

[CR52] Pollack RM, Donath MY, LeRoith D, Leibowitz G (2016). Anti-inflammatory agents in the treatment of diabetes and its vascular complications. Diabet Care.

[CR53] Pondei K, Fente BG, Oladapo O (2013). Current microbial isolates from wound swabs, their culture and sensitivity pattern at the Niger Delta University Teaching Hospital, Okolobiri. Nigeria Trop Med Health.

[CR54] Rani PR, Degati D, Lakshmi DV, Vijaya D, Prasad R (2019) Lytic bacteriophages and phage cocktails seems to be a future alternative against multi-drug resistant bacterial infections. Preprint 2019:1–64

[CR55] Roach DR, Debarbieux L (2017). Phage therapy: awakening a sleeping giant. Emerg Top Life Sci.

[CR56] Saltoglua N, Ergonulb O, Tulekc N, Yemisena M, Kadanalid A, Karagozd G, Batirele A (2018). Influence of multidrug resistant organisms on the outcome of diabetic foot infection. Int J Infec Dis.

[CR57] Sanchez CJ, Mende K, Beckius ML, Akers KS, Romano DR, Wenke JC, Murray C (2013). Biofilm formation by clinical isolates and the implications in chronic infections. BMC Infect Dis.

[CR58] Schmittgen TD, Livak KJ (2008). Analyzing real-time PCR data by the comparative CT method. Nat Protocol.

[CR59] Shang A, Liu Y, Wang J, Mo Z, Li G, Mou H (2015). Complete nucleotide sequence of *Klebsiella* phage P13 and prediction of an EPS depolymerase gene. Virus Genes.

[CR60] Shanmugam P, Jeya M, Linda SS (2013). The bacteriology of diabetic foot ulcers, with a special reference to multidrug resistant strains. J Clin Diagn Res.

[CR61] Shi Y, Shu B, Yang R, Xu Y, Xing B, Liu J, Wang P, Tang J, Xie J (2015). Wnt and Notch signaling pathway involved in wound healing by targeting separately c-Myc and Hes1. Stem Cell Res Therapy.

[CR62] Shigehisa R, Uchiyama J, Kato SI, Takemura-Uchiyama I, Yamaguchi K, Miyata R, Ujihara T, Sakaguchi Y, Okamoto N, Shimakura H, Daibata M, Sakaguchi M, Matsuzaki S (2016). Characterization of *Pseudomonas aeruginosa* phage KPP21 belonging to family Podoviridae genus N4-like viruses isolated in Japan. Microbiol Immunol.

[CR63] Solarek W, Koper M, Lewicki S, Szczylik C, Czarnecka AM (2019). Insulin and insulin-like growth factors act as renal cell cancer intratumoral regulators. J Cell Comm Signal.

[CR64] Son JS, Lee SJ, Jun SY, Yoon SJ, Kang SH, Paik HR, Kang JO, Choi YJ (2010). Antibacterial and biofilm removal activity of a podoviridae *Staphylococcus aureus* bacteriophage SAP-2 and a derived recombinant cell-wall-degrading enzyme. Appl Microbiol Biotechnol.

[CR65] Soothill JS (1992). Treatment of experimental infections of mice with bacteriophages. J Med Microbiol.

[CR66] Speck P, Smithyman A (2016). Safety and efficacy of phage therapy via the intravenous route. FEMS Microbiol Lett.

[CR67] Spichler A, Hurwitz B, David GA, Lipsky BA (2015). Microbiology of diabetic foot infections: from Louis Pasteur to ‘crime scene investigation’. BMC Med.

[CR68] Sulakvelidze A, Alavidze Z, Morris JG (2001). Bacteriophage therapy. Antimicrob Agents Chemother.

[CR69] Suvik A, Effendy AWM (2012). The use of modified Masson’s trichrome staining in collagen evaluation in wound healing study. Mal J Vet Res.

[CR70] Sweere JM, Van Belleghem JD, Ishak H, Bach MS, Popescu M, Sunkari V, Kaber G, Manasherob R, Suh GA, Cao X (2019). Bacteriophage trigger antiviral immunity and prevent clearance of bacterial infection. Sci.

[CR71] Teng T, Li Q, Liu Z, Li X, Liu Z, Liu H, Liu F, Xie L, Wang H, Zhang L, Wu D, Chen M, Li Y, Ji A (2019). Characterization and genome analysis of novel *Klebsiella* phage Henu1 with lytic activity against clinical strains of *Klebsiella pneumoniae*. Arch Virol.

[CR72] Van Belleghem JD, Clement F, Merabishvili M, Lavigne R, Vaneechoutte M (2017). Pro-and anti-inflammatory responses of peripheral blood mononuclear cells induced by *Staphylococcus aureus* and *Pseudomonas aeruginosa* phages. Sci Report.

[CR73] Xavier W, Sukumaran MT, Varma AK, Kumar H, Chellan G (2014). Emergence of multi drug resistant bacteria in diabetic patients with lower limb wounds. Ind J Med Res.

[CR74] Zakaria AS, Afifi SA, Elkhodairy KA (2016). Newly developed topical cefotaxime sodium hydrogels: antibacterial activity and *in vivo* evaluation. BioMed Res Int.

[CR75] Zaman SB, Hussain MA, Nye R, Mehta V, Mamun KT, Hossain N (2017). A review on antibiotic resistance: alarm bells are ringing. Cureus.

[CR76] Zhang M, Jiang Z, Li D, Jiang D, Wu Y, Ren H, Peng H, Lai Y (2014). Oral antibiotic treatment induces skin microbiota dysbiosis and influences wound healing. Micro Ecol.

[CR77] Zhang Z, Jiang D, Wang C, Garzotto M, Kopp R, Wilmot B, Thuillier P, Dang A, Palma A, Farris PE, Shannon J (2019). Polymorphisms in oxidative stress pathway genes and prostate cancer risk. Cancer Causes Cont.

