# Supplementary material for: Bacteriophage therapy as an alternative technique for treatment of multidrug-resistant bacteria causing diabetic foot infection
Source: Int Microbiol. 2022 Nov 9;26(2):343–59. doi: 10.1007/s10123-022-00293-2 (PMC10148765; doi:10.1007/s10123-022-00293-2)
Supplement: Supplementary file 1 — Supplementary file1 (DOCX 928 KB) [file 10123_2022_293_MOESM1_ESM.docx]

Sup 1. Primers used for bacterial identification and determination of wound gene expression.

| Gene | Forward primer  (5′–3′) | Reverse primer  (5′–3′) | Size(bp) | Accession  No. |
| --- | --- | --- | --- | --- |
| PCNA | ATCTAGACGTCGCAACTCCG | GCTGCACTAAGGAGACGTGA | 173 | [NM_022381.3](https://www.ncbi.nlm.nih.gov/entrez/viewer.fcgi?db=nucleotide&id=148747129) |
| Mmp9 | GATCCCCAGAGCGTTACTCG | GTTGTGGAAACTCACACGCC | 132 | [NM_031055.2](https://www.ncbi.nlm.nih.gov/entrez/viewer.fcgi?db=nucleotide&id=1937369759) |
| Collagen | GCAATGCTGAATCGTCCCAC | CAGCACAGGCCCTCAAAAAC | 176 | [NM_053304.1](https://www.ncbi.nlm.nih.gov/entrez/viewer.fcgi?db=nucleotide&id=158711703) |
| Fibronectin | GGATCCCCTCCCAGAGAAGT | GGGTGTGGAAGGGTAACCAG | 188 | [NM_019143.2](https://www.ncbi.nlm.nih.gov/entrez/viewer.fcgi?db=nucleotide&id=186972113) |
| NF-κβ1 | CCACTGTCAACAGATGGCCC | CTTTGCAGGCCCCACATAGT | 177 | [NM_001276711.1](https://www.ncbi.nlm.nih.gov/entrez/viewer.fcgi?db=nucleotide&id=453040304) |
| TNF-α | GGCTTTCGGAACTCACTGGA | GGGAACAGTCTGGGAAGCTC | 164 | [NM_012675.3](https://www.ncbi.nlm.nih.gov/entrez/viewer.fcgi?db=nucleotide&id=260166688) |
| IL-10 | GCTCAGCACTGCTATGTTGC | TTGTCACCCCGGATGGAATG | 76 | [NM_012854.2](https://www.ncbi.nlm.nih.gov/entrez/viewer.fcgi?db=nucleotide&id=148747381) |
| IL-8 | ACAGGCAGGCTGTAGTTGTC | ATCACCAGCGAGTTTCCCAG | 70 | [NM_019310.1](https://www.ncbi.nlm.nih.gov/entrez/viewer.fcgi?db=nucleotide&id=9506808) |
| IL-4 | CGTGATGTACCTCCGTGCTT | GTGAGTTCAGACCGCTGACA | 88 | [NM_201270.1](https://www.ncbi.nlm.nih.gov/entrez/viewer.fcgi?db=nucleotide&id=42627876) |
| IL-1β | GAGTCTGCACAGTTCCCCAA | TCCTGGGGAAGGCATTAGGA | 158 | [NM_031512.2](https://www.ncbi.nlm.nih.gov/entrez/viewer.fcgi?db=nucleotide&id=158186735) |
| MCP-1 | TAGCATCCACGTGCTGTCTC | CAGCCGACTCATTGGGATCA | 94 | [NM_031530.1](https://www.ncbi.nlm.nih.gov/entrez/viewer.fcgi?db=nucleotide&id=13928713) |
| TGF- β1 | AGGGCTACCATGCCAACTTC | CCACGTAGTAGACGATGGGC | 168 | [NM_021578.2](https://www.ncbi.nlm.nih.gov/entrez/viewer.fcgi?db=nucleotide&id=148747597) |
| IL-1β | GAGTCTGCACAGTTCCCCAA | TCCTGGGGAAGGCATTAGGA | 158 | [NM_031512.2](https://www.ncbi.nlm.nih.gov/entrez/viewer.fcgi?db=nucleotide&id=158186735) |
| Gapdh | GCATCTTCTTGTGCAGTGCC | GGTAACCAGGCGTCCGATAC | 91 | [NM_017008.4](https://www.ncbi.nlm.nih.gov/entrez/viewer.fcgi?db=nucleotide&id=402691727) |

Sup. 2. Identification of isolated bacteria by Vitek2 GP card.

| Bacterial isolates | | | | | | | | Biochemical tests | No. of tests |
| --- | --- | --- | --- | --- | --- | --- | --- | --- | --- |
| *E. cloacae* | *A. baumannii* | *E. coli* | *P. mirabilis* | *K.pneumonia* | *P. aeruginosa* | *E. faecalis* | *S. aureus* |  |  |
| ND | ND | ND | ND | ND | ND | + | - | D- Amygladin (AMY) | 1 |
| - | - | - | - | - | - | - | - | Ala-Phe-Pro Arylamidase (APPA) | 2 |
| ND | ND | ND | ND | ND | ND | - | - | Leucine Arylamidase (LeuA) | 3 |
| ND | ND | ND | ND | ND | ND | + | + | D- Ribose (dRIB) | 4 |
| ND | ND | ND | ND | ND | ND | + | + | Novobiocin Ristance (NOVO) | 5 |
| ND | ND | ND | ND | ND | ND | - | - | D-Raffinose (dRAF) | 6 |
| ND | ND | ND | ND | ND | ND | + | + | Optochin Resistance (OPTO) | 7 |
| ND | ND | ND | ND | ND | ND | - | - | Phospatidyle- Nosit phospholipase C (PIPLC) | 8 |
| ND | ND | ND | ND | ND | ND | + | - | Cyclodextrin (CDEX) | 9 |
| + | - | + | - | - | + | - | - | L-Proline Arylamidase (ProA) | 10 |
| - | + | + | - | + |  | + | - | Tyrosine Arylamidase (TyrA) | 11 |
| + | + | + | - | + | + | + | + | L-Lactate Alkalinization (lLATk) | 12 |
| ND | ND | ND | ND | ND | ND | + | + | Growth in 1.1% Nacl (NC1.1) | 13 |
| + | + | + | + | + | - | + | + | O/129 Resistanec (Comp. Vibrio) O129R | 14 |
| ND | ND | ND | ND | ND | ND | - | - | D-Xylose (dxYL) | 15 |
| ND | ND | ND | ND | ND | ND | + | - | L aspartate Arylamidase (AsPA) | 16 |
| ND | ND | ND | ND | ND | ND | - | - | Beta- glucuronidase (BGURr) | 17 |
| + | - | + | - | + | - | - | - | D-Sorbitol ( dSOR) | 18 |
| ND | ND | ND | ND | ND | ND | - | - | Lactose (lAC) | 19 |
| + | - | + | - | + | + | + | - | D-Mannitol (dMAN) | 20 |
| ND | ND | ND | ND | ND | ND | + | - | SALICIN (SAL) | 21 |
| ND | ND | ND | ND | ND | ND | + | - | Alanine- Arylamidase (Ala A) | 22 |
| ND | ND | ND | ND | ND | ND | + | + | Arginine Dihydrolase (ADH1) | 23 |
| ND | ND | ND | ND | ND | ND | - | - | Beta- Galacopyronidase (BGAR) | 24 |
| + | - | + | - | + | - | - | - | Alpha- Galactosidase (AGAL) | 25 |
| - | - | - | + | - | - | + | + | Urease (URE) | 26 |
| ND | ND | ND | ND | ND | ND | + | + | N-Acetyle- D-Glucosamine (NAG) | 27 |
| ND | ND | ND | ND | ND | ND | + | + | D-Mannose (dMNE) | 28 |
| + | - | - | - | + | - | + | + | Saccharose-Sucrose (SAC) | 29 |
| ND | ND | ND | ND | ND | ND | - | - | Beta- Galactosidase (BGAL) | 30 |
| ND | ND | ND | ND | ND | ND | - | - | Alpha- Mannosidase (AMAN) | 31 |
| - | + | - | - | + |  | + | + | L- Pyrrolidonyl- Arylamidase (PyrA) | 32 |
| ND | ND | ND | ND | ND | ND | + | + | Polymyxin b Resistance (POLYB) | 33 |
| + | - | + | - | + | - | + | + | D- Maltose (dMAL) | 34 |
| ND | ND | ND | ND | ND | ND | + | + | Methyl-B-D-Glucopyranoside (MBdG) | 35 |
| ND | ND | ND | ND | ND | ND | + | + | D-trehalose (dTRE) | 36 |
| ND | ND | ND | ND | ND | ND | - | + | Alpha-Glucosidases (AGLU) | 37 |

Sup. 2, continued

| bacterial isolates | | | | | | | | Biochemical tests | No.  of test |
| --- | --- | --- | --- | --- | --- | --- | --- | --- | --- |
| *E. cloacae* | *A. baumannii* | *E. coli* | *P. mirabilis* | *K.pneumonia* | *P. aeruginosa* | *E. faecalis* | *S. aureus* |  |  |
| - | - | - | + | + | - | + | + | Phosphatase (PHOS) | 38 |
| - | - | - | - | - | - | - | - | B-Glucoronidase (BGUR) | 39 |
| ND | ND | ND | ND | ND | ND | - | - | D-Galactose (dGAL) | 40 |
| ND | ND | ND | ND | ND | ND | + | + | Bacitracin Resistance (BACL) | 41 |
| ND | ND | ND | ND | ND | ND | - | - | Pullulan (PUL) | 42 |
| ND | ND | ND | ND | ND | ND | + | - | Arginine Dihydrolase 2 (ADH2S) | 43 |
| - | - | - | - | - | + | ND | ND | Lipase (LIP) | 44 |
| - | - | - | - | - | + | ND | ND | Tagatose (dTAG) | 45 |
| - | - | - | - | - | - | ND | ND | Alpha Glocosidase (AGLU) | 46 |
| + | - | - | + | - | - | ND | ND | Ornithine decarboxylase (ODC) | 47 |
| - | + | - | - | - | + | ND | ND | GlutamyleArylamidaseNA (AGLTP) | 49 |
| + | - | - | - | + | - | ND | ND | Palatinose (PLE) | 50 |
| + | - | + | + | + | - | ND | ND | D- trehalose (dTRE) | 51 |
| - | + | + | - | + | + | ND | ND | Succinate Alkanization (SUCT) | 52 |
| - | - | + | - | + | - | ND | ND | Lysine Decarboxylase (LDC) | 53 |
| + | + | + | + | + | + | ND | ND | D- Glucose (dGlucose) | 54 |
| + | + | + | - | + | + | ND | ND | d- Mannose (dMNE) | 55 |
| + | + | - | - | + | + | ND | ND | CIT citrate (sodium) | 56 |
| + | - | - | - | - | - | ND | ND | Beta N-Acetyle (NAGA) | 57 |
| - | + | - | - | + | - | ND | ND | L-Histidine assimilation (IHISa) | 58 |
| + | - | - | - | + | + | ND | ND | Gamma-Glutamyle-Transferase (GGT) | 59 |
| + | - | - | - | + | - | ND | ND | Beta- Xylosidase (BXYL) | 60 |
| + | + | - | - | + | + | ND | ND | Malonate (MNT) | 61 |
| + | - | + | - | + | - | ND | ND | Alpha – Glactosidase (AGAL) | 62 |
| - | - | + | - | + | - | ND | ND | Beta Glactosidase (BGAL) | 63 |
| + | - | + | + | + | - | ND | ND | Fermentation/ Glucose (OFF) | 64 |
| + | - | - | - | - | + | ND | ND | Beta-Alanine Arylamidase PNA (BAlap) | 65 |
| - | - | + | - | - | - | ND | ND | 5-keto-D-Gluconate (5KG) | 66 |
| - | - | - | + | - | - | ND | ND | Hydrogen Sulphide (H2S) | 67 |
| - | - | - | - | + | - | ND | ND | Beta-Glucosidase (BGLU) | 68 |
| + | - | + | - | - | - | ND | ND | Glycine Aryamidase (GlyA) | 69 |

*+ = Positive, - = negative, ND= Not detected.


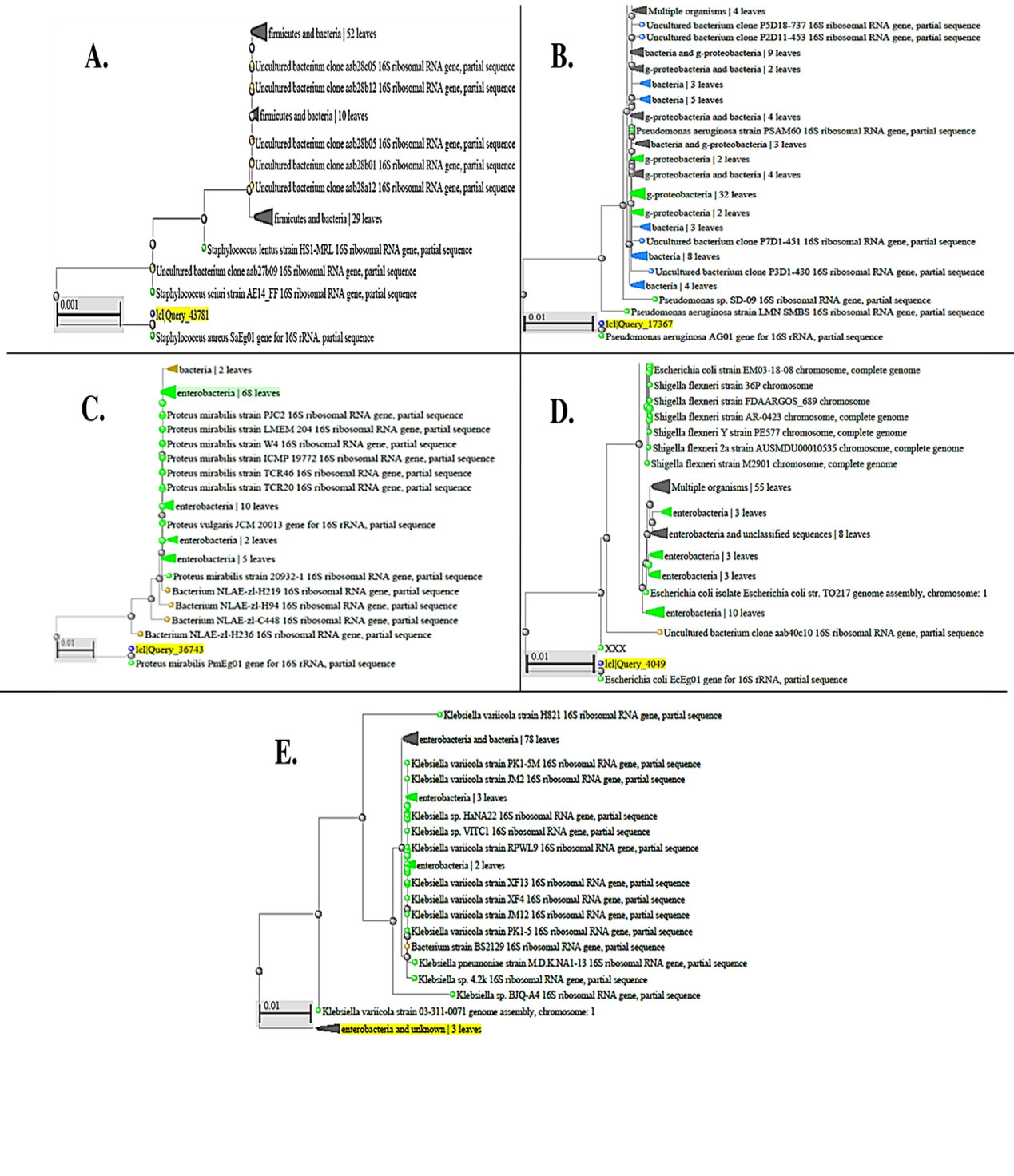


Sup.3. Phylogenetic tree analysis based on 16 s rRNA nucleotide sequence alignment for five clinical isolates with other related members that possess the best similarity (A.:E.). A. Fig. (10): Phylogenetic tree analysis based on 16 s rRNA nucleotide sequence alignment for *Staphylococcus aureus* with some other related members that possess the best similarity, B. Phylogenetic tree analysis based on 16 s rRNA nucleotide sequence alignment for *Pseudomonas aeruginosa* with some other related members that possess the best similarity, C. Phylogenetic tree analysis based on 16 s rRNA nucleotide sequence alignment for *Proteus mirabilis* with some other related members that possess the best similarity., D. Phylogenetic tree analysis based on 16 s rRNA nucleotide sequence alignment for *Escherichia coli* with some other related members that possess the best similarity, and E. Phylogenetic tree analysis based on 16 s rRNA nucleotide sequence alignment for *Klebsiella variicola* with some other related members that possess the best similarity.


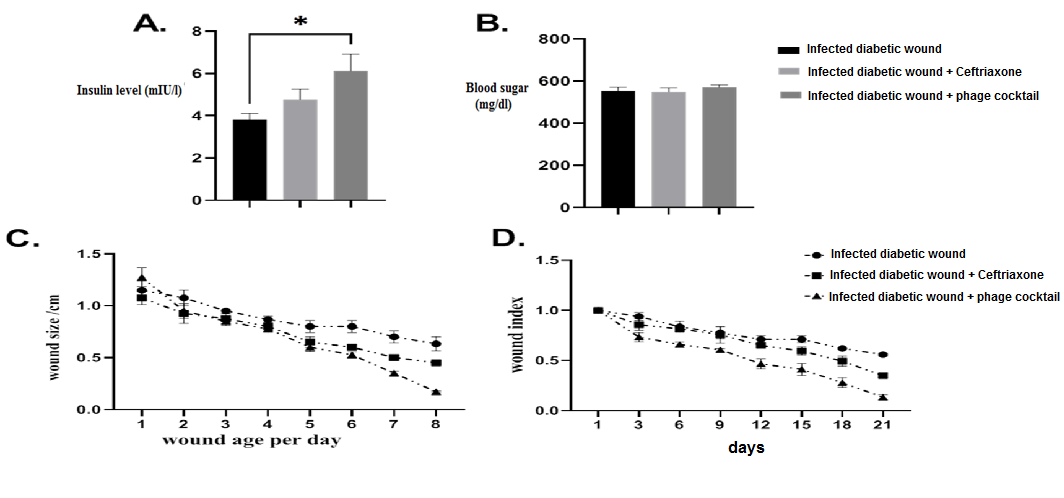


Sup. 4. Glycemic (A. & B.) and wound parameters (C, D) of type1 diabetic rats throughout 21 days, (A.) fasting blood glucose level (mg/dl), (B.) serum insulin level (mIU/l), (C.) wound diameter (cm) and (D.) wound index.
